# Characterizing the metabolic effects of the selective inhibition of gut microbial β-glucuronidases in mice

**DOI:** 10.1038/s41598-022-21518-4

**Published:** 2022-10-19

**Authors:** Marine P. M. Letertre, Aadra P. Bhatt, Michael Harvey, Jeremy K. Nicholson, Ian D. Wilson, Matthew R. Redinbo, Jonathan R. Swann

**Affiliations:** 1 Department of Metabolism, Digestion and Reproduction, Imperial College London, London, UK; 2 CNRS, CEISAM, UMR 6230, Nantes Université, 44000 Nantes, France; 3 Department of Medicine, University of North Carolina, Chapel Hill, NC 27599 USA; 4 School of Human Development and Health, Faculty of Medicine, University of Southampton, Southampton, UK; 5 The Australian National Phenome Centre, Health Futures Institute, Murdoch University, Perth, Australia; 6 Institute of Global Health Innovation, Faculty of Medicine, Imperial College London, London, UK; 7 Departments of Chemistry, Biocemistry, Microbiology and Genomics, University of North Carolina, Chapel Hill, NC 27599 USA

**Keywords:** Metabolomics, Toxicology, Microbiome

## Abstract

The hydrolysis of xenobiotic glucuronides by gut bacterial glucuronidases reactivates previously detoxified compounds resulting in severe gut toxicity for the host. Selective bacterial β-glucuronidase inhibitors can mitigate this toxicity but their impact on wider host metabolic processes has not been studied. To investigate this the inhibitor 4-(8-(piperazin-1-yl)-1,2,3,4-tetrahydro-[1,2,3]triazino[4′,5′:4,5]thieno[2,3-c]isoquinolin-5-yl)morpholine (UNC10201652, Inh 9) was administered to mice to selectively inhibit a narrow range of bacterial β-glucuronidases in the gut. The metabolomic profiles of the intestinal contents, biofluids, and several tissues involved in the enterohepatic circulation were measured and compared to control animals. No biochemical perturbations were observed in the plasma, liver or gall bladder. In contrast, the metabolite profiles of urine, colon contents, feces and gut wall were altered compared to the controls. Changes were largely restricted to compounds derived from gut microbial metabolism. This work establishes that inhibitors targeted towards bacterial β-glucuronidases modulate the functionality of the intestinal microbiota without adversely impacting the host metabolic system.

## Introduction

The intestinal microbiome contains an estimated 3 million different microbial genes, many of which encode enzymes that perform metabolic functions absent from the mammalian host genome^1^. As such, the gut microbiome expands the biotransformational capabilities available to the host, increasing the dietary substrates and xenobiotics that can be processed, and broadens the diversity of molecules to which the host is exposed. One class of enzymes encoded in the intestinal metagenome are the bacterial β-glucuronidases (GUS) that hydrolyze glucuronic acid from dietary components and host-derived conjugates excreted into the gut via the bile. This glucuronic acid provides bacteria with an energy source when converted to glyceraldehyde-3-phosphate and pyruvate, which then enters glycolysis^2^. Over 3,000 different β-glucuronidases have been described in fecal samples from 139 human donors^3^, with inter-individual variability reported in terms of the number of genes encoding for these enzymes and also in terms of their functionality across the different β-glucuronidases. A similar cadre of microbial β-glucuronidases have been identified in the mouse gut microbiome^4^.

Microbial glucuronidases have been known for many years to remove the glucuronic acid from xenobiotics inactivated by conjugative metabolism and excreted into the gut via the bile. This activity releases the active compound within the gastrointestinal tract, which can lead to toxicity^5^. An example of this is the chemotherapeutic irinotecan which is activated by hydroxylation in the liver, producing the active metabolite SN-38, which is subsequently inactivated and detoxified by glucuronidation. This glucuronide conjugate is then excreted into the gut, via the bile, where bacterial β-glucuronidase activity can release the active, and toxic, SN-38, causing severe intestinal toxicity that can result in the discontinuation of treatment^6,7^. With the increasing interest in gut commensal bacterial β-glucuronidase proteins, chemical inhibitors of these enzymes have been developed^8^. In mice, the administration of these inhibitors has been shown to prevent irinotecan toxicity^8–10^. Similarly, NSAID glucuronide conjugates are often excreted via the bile and their subsequent hydrolysis releases the active drug, which can again result in gut toxicity. This toxicity can be prevented by the chemical inhibitor, Inh-1 ([1-((6,8-dimethyl-2-oxo-1,2-dihydroquinolin-3-yl)-3-(4-ethoxyphenyl)-1-(2- hydroxyethyl) thiourea])^8,11,12^. Inh-1 has also been shown to protect rats from NSAID-induced anastomotic leakage after intestinal surgery^13^.

In this study we characterized the biochemical changes that occur to the murine metabolome of various different “compartments” following the targeted inhibition of gut microbial β-glucuronidase enzymes within treated and control mice. The inhibitor selected was UNC10201652 (Inh-9, [4-(8-(piperazin-1-yl)-1,2,3,4-tetrahydro-[1,2,3]triazino[4′,5′:4,5] thieno[2,3-c]isoquinolin-5-yl)morpholine]), which has been shown to uniquely hijack the catalytic cycle of gut microbial GUS enzymes^14^. We have previously demonstrated that this inhibitor can reach the small and large intestine intact, has a negligible impact on the diversity and composition of the gut microbiota^10,15^ and did not alter the murine host phenotype, including body weight, colon length and structure, or result in colonic or systematic inflammation^10,15^. However, these studies, undertaken to explore the effectiveness of UNC10201652 as a means of limiting the toxicity of drugs such as irinotecan and NSAID’s, shed little light on the potential of the inhibitor itself to disrupt both host and gut microbiota metabolism. Understanding the wider implications of the pharmacology of this inhibitor on the metabolic state of the overall holobiont is important to assess its potential as a strategy to attenuate microbiota-related drug toxicity.

## Results

Inhibition was achieved through three daily oral doses of UNC10201652 (20 μg/mouse), administrated at 0, 24 and 48 h, a regime which has been previously demonstrated to reduce gut bacterial GUS activity^10^. Tissues throughout the enterohepatic circulation (colon luminal contents, colon wall, liver, gall bladder) as well as plasma reflecting the systemic circulation, were collected 96 h after the first dose. Urine and feces, reflecting routes of excretion, were collected at pre-dose, and 24, 48 and 96 h after receiving the first dose. ^1^H Nuclear magnetic resonance (NMR) spectroscopy and ultra-performance liquid chromatography-mass spectrometry (UPLC-MS) were used to obtain untargeted metabolic profiles of these samples.

### Plasma samples

Unsupervised analyses performed on the ^1^H NMR spectroscopy dataset for the plasma samples showed no sign of clustering by treatment group (Control *n* = 7; Treated *n* = 8; Supplementary Figure S1). Supervised covariate-adjusted projection to latent structures-discriminant analysis (CA-PLS-DA) and Wilcoxon rank sum test were used to analyze the significance of the treatment group separation along the predictive component of the models. No significant differences (α < 0.05) were identified between the groups for the plasma samples measured by ^1^H NMR spectroscopy (Supplementary Figure S2).

### Urine samples

**Figure 1:**
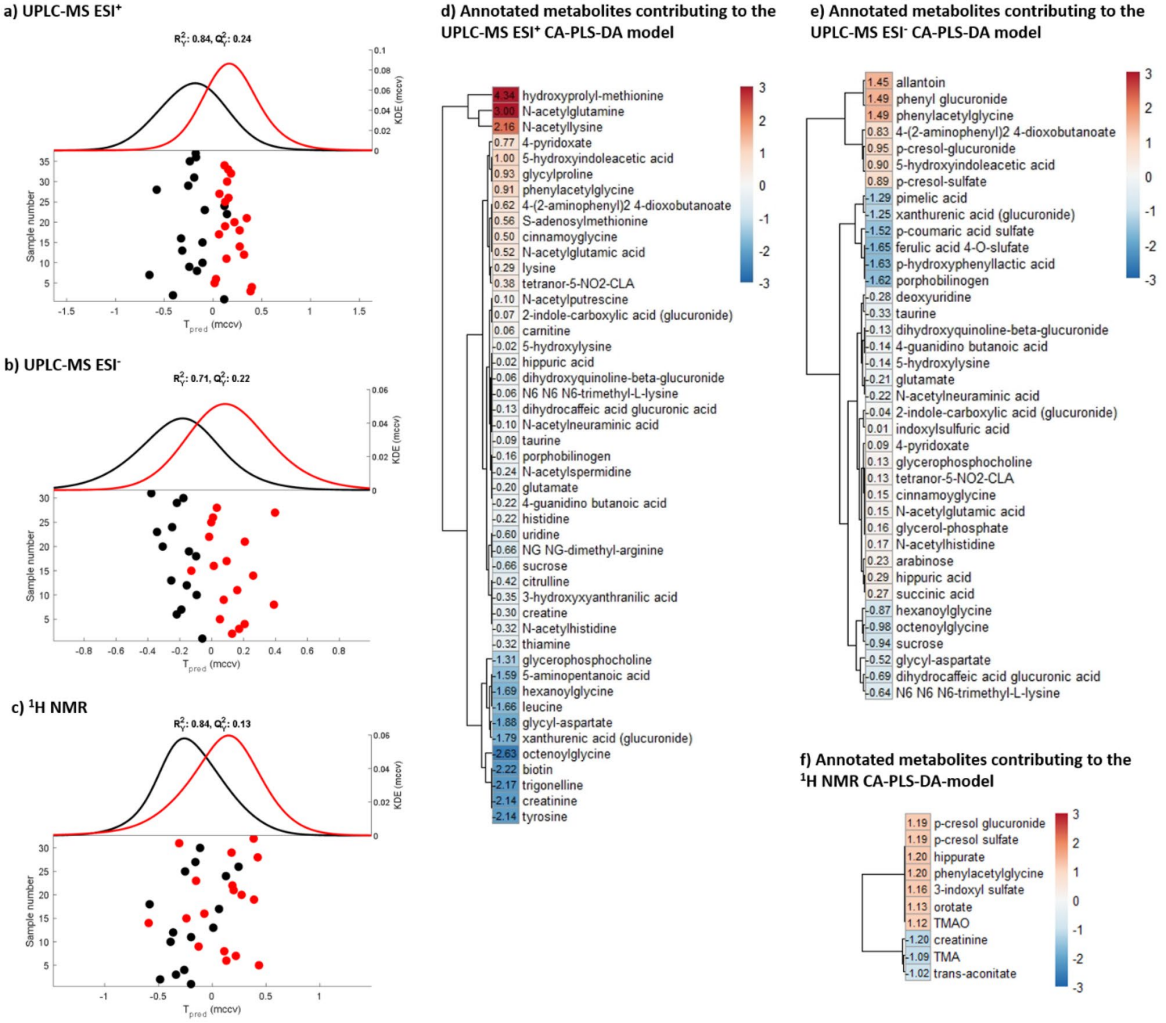
Urine metabolic profiling of the UNC10201652 treated animals compared to control. CA-PLS-DA scores plots with kernel density estimation obtained from (**a**) UPLC-MS ESI^+^ (*P* = 2.67 × 10^–6^), (**b**) UPLC-MS ESI^−^ (*P* = 4.55 × 10^–6^) and (**c**) ^1^H NMR spectroscopic (*P* = 0.005) datasets. Each point represents an individual sample. All the samples available at the different post-dose time points (24, 48 and 96 h following the first dose of UNC10201652) have been considered to ensure statistical robustness. Black points represent the control animals and the red points the treated animals. Heatmap of the putatively annotated metabolites found significantly different between the UNC10201652-treated animals and the control animals, in the urine samples by (**d**) ^1^H NMR spectroscopy, (**e**) UPLC-MS ESI^+^ and (**f**) ESI^–^. The colors and the values shown in the heatmap represent the − log_10_(q values) × the Manhattan sign extracted from the CA-PLS-DA models. These visualize the urinary metabolites found to increase (red) or decrease (blue) in the animals that received the inhibitor treatment compared to the control animals.

PCA models built on the urinary metabolic profiles measured by ^1^H NMR spectroscopy and UPLC-MS identified that sex was a major source of metabolic variation (Supplementary Figure S3). After adjusting for sex, significant CA-PLS-DA models were obtained comparing the urinary metabolic profiles of the control and treated mice. Significance was observed for both ^1^H NMR spectral data (*P* = 0.005) and the UPLC-MS ESI^+^ (*P* = 2.67 × 10^–6^) and the UPLC-MS ESI^−^ (*P* = 4.55 × 10^–6^) datasets (Fig. 1).

From the ^1^H NMR spectral data, mice treated with the GUS inhibitor were found to excrete greater amounts of trimethylamine-*N*-oxide (TMAO), 3-indoxyl sulfate (3-IS), phenylacetylglutamine (PAG), orotate, hippurate, *p*-cresyl sulfate (*p*CS) and *p*-cresyl glucuronide (*p*CG) compared to the control mice and lower amounts of creatinine, trimethylamine (TMA) and *trans*-aconitate. The UPLC-MS ESI^−^ data also showed greater amounts of PAG, *p*CS and *p*CG in the urine of the treated mice, as well as greater amounts of phenyl glucuronide and allantoin. In the ESI^+^ dataset, the excretion of hydroxyprolyl-methionine, *N*-acetyl glutamine and *N*-acetyl lysine was noted to be significantly higher in the urine of treated mice compared to that of vehicle dosed controls. In contrast, several compounds were found to be excreted in lower amounts by the treated animals, including uridine, leucine, NG,NG-dimethyl-arginine, tyrosine, *N*-methylnicotinic acid (NMNA, trigonelline), biotin, hexanoylglycine and octenoylglycine, glycyl-aspartate, and 3-hydroxyanthranilic acid.

Several glucuronides were identified in the ESI^+^ dataset to be modulated by inhibitor intake. The excretion of dihydroxyquinoline-β-glucuronide (dihydrocaffeic acid glucuronide) was lower in mice receiving the inhibitor compared to the control mice. Moreover, two other glucuronides were tentatively annotated by the neutral loss of 176 Da from the [M+H]^+^ ion. These putative glucuronides were 2-indole-carboxylic acid glucuronide, which was higher in the urine of treated mice, and xanthurenic acid glucuronide, which was lower in the urine samples from treated compared to control mice. Consistent with the positive ionization mode data, the excretion of the dihydroxyquinoline, dihydrocaffeic acid and xanthurenic acid glucuronides, observed with the negative ionization mode, was lower in mice receiving the inhibitor compared to those receiving the saline control. In negative ESI the amounts of some sulfate-conjugated metabolites were also observed to have been altered by exposure to the inhibitor UNC10201652, including *p*CS, whose excretion was significantly greater in the treated group, whilst the relative amounts of *p*-coumaric acid sulfate and ferulic acid 4-*O-*sulfate were lower. Another metabolite from tyrosine catabolism, *p*-hydroxyphenyllactic acid, was excreted in lower amounts following inhibitor treatment. Also present in lower amounts in urine from treated mice, as detected using negative ESI, were sucrose, glycyl-aspartate, porphobilinogen, hexanoylglycine and octenoylglycine (similarly to the positive ESI) and pimelic acid. In contrast 4-pyridoxate, cinnamoyglycine, *N*-acetyl glutamic acid (similarly to the positive ESI data), glycerol-phosphate, *N*-acetyl histidine, arabinose, and succinate, were present in greater amounts. The fatty acyl tetranor-5-NO_2_-CLA was increased in the urine samples of the treated mice and detected in both ionization modes. More detailed information on the mas spectral data aquired on these putatively annotated features is provided in Table S2 (positive ESI) and Table S3 (negative ESI).

### Fecal samples

**Figure 2:**
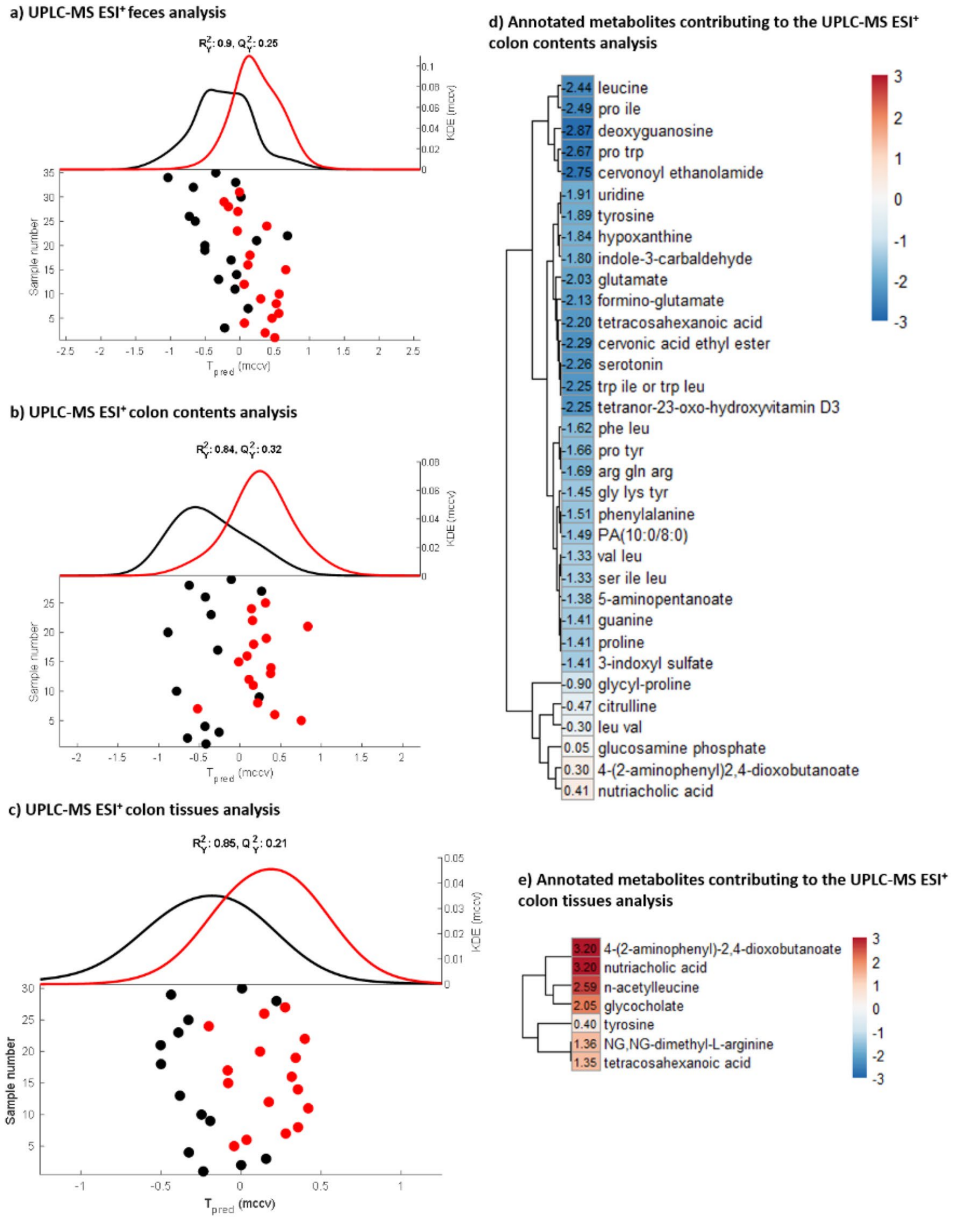
Fecal, colon tissue and colon content metabolic profiling of UNC10201652 treated animals compared to control. CA-PLS-DA scores plots with kernel density estimation from the models built on the metabolic profiles measured by UPLC-MS ESI^+^ for the (**a**) fecal samples (*P* = 7.17 × 10^–4^), (**b**) colon contents, (*P* = 5.76 × 10^–4^) and c) colon tissues (*P* = 2.75 × 10^–4^). Each point represents an individual sample. For the fecal samples, all the samples available at the different post-dose time points (24, 48 and 96 h following the first dose of UNC10201652) have been included to ensure statistical robustness. For the colon tissues and the colon contents samples which were collected only after animal euthanasia, the time point considered is 96 h following the first dose of the inhibitor. This included technical replicates which were adjusted for in the model. Black points represent the control animals and the red points the treated animals. (**b**) Heatmaps of the annotated metabolites identified to differ between the groups in the colon contents and the colon tissues. Colors and values of the heatmap represent the − log_10_(q values) × the Manhattan sign obtained in the CA-PLS-DA models, visualizing metabolites that increased (red) or decreased (blue) in the animals that received the inhibitor treatment compared to the control animals.

A significant difference was observed between GUS inhibitor-treated and untreated mice in the CA-PLS-DA model built on the UPLC-MS ESI^+^ dataset from the fecal samples (*P* = 7.17 × 10^–4^, Fig. 2 and Supplementary Figure S4). The features in the feces found to be discriminatory between the control and treated mice were all lipids, particularly sterol lipids (data not shown). However, as the UPLC-MS RP method applied was not optimized for this class of metabolites, confident annotation was not possible. No differences were detected in the fecal profiles measured by UPLC-MS using negative ESI.

### Colon contents

Metabolic profiles of the colonic contents reflect the metabolites present in the lumen of the colon. No significant variation was observed in the UPLC-MS ESI^−^ metabolic profiles of the colon contents between the treatment groups (Figure S4) by PCA. However, a significant difference was observed for the CA-PLS-DA model built on the UPLC-MS ESI^+^ dataset (*P* = 5.76 × 10^–4^, Fig. 2). UNC10201652-treated animals generally had a lower abundance of features with a *m*/*z* between 50 and 600, eluting up to 8 min into the analysis (*N* = 255), and an increase in features having a *m*/*z* ~ 650 Da eluting after 8 min (*N* = 57). Putative annotation of the discriminatory features indicated that inhibitor-dosed mice had reduced amounts of the fatty-acid tetracosahexaenoic acid (which was increased in colon tissues), phosphatidic acid (10:0/8:0), and the vitamin D derivative tetranor-23-oxohydroxyvitamin D3 compared to the control mice. Similarly, hypoxanthine, leucine, deoxyguanosine, uridine, tyrosine, glutamate, formino-glutamate, indole-3-carbaldehyde, indole acrylic acid, and serotonin were less abundant in the colon contents of treated animals compared to the control group. Several dipeptides and tripeptides were present in lower amounts in the samples from treated mice, including glycyl-proline (greater in the urine of treated animals), prolyl-tyrosine, leucyl-valine, prolyl-isoleucine, glycyl-lysyl-tyrosine, seryl-isoleucyl-leucine, phenylalanyl-leucine, prolyl-tryptophan, arginyl-glutamyl-arginine, and tryptohan-leucine (Fig. 2). Conversely, the colon contents of treated mice contained greater amounts of nutriacholic acid, 4-(2-aminophenyl)-2,4-dioxobutanoate (also more abundant in the urine of these animals), and glucosamine phosphate. More detailed information on the mass spectral data aquired on these putatively annotated features is provided in Table S4.

### Colon tissues

For the colon tissue extracts, no separation was observed between the treatment groups based on the unsupervised analyses performed on the ^1^H NMR spectroscopic and UPLC-MS datasets (Supplementary Figure S4). Supervised CA-PLS-DA analysis in combination with the Wilcoxon rank sum test showed that there were no significant differences (α < 0.05) between the colon profiles from the two groups in either the ^1^H NMR spectroscopic or the UPLC-MS ESI^−^ datasets (Supplementary Figure S5). However, a significant difference was observed for the UPLC-MS ESI^+^ dataset of the colon tissue extracts (*P* = 2.75 × 10^–4^, Fig. 2). Features in the positive ESI dataset found to be significantly different between the treated and control mice were putatively annotated (Table S5). Colon tissue from the treated animals contained higher amounts of 4-(2-aminophenyl)-2,4-dioxobutanoate, NG,NG-dimethyl-arginine (found to be reduced in urine), *N*-acetyl leucine, the fatty acid tetracosahexanoic acid, and the bile acids, nutriacholic acid and glycocholic acid, compared to control tissue. Tyrosine content was also observed to be moderately increased in the colon tissue of treated compared to the controls, whilst being lower in urine.

### Liver tissues

Neither unsupervised nor supervised analyses performed on the ^1^H NMR profiles or the UPLC-MS datasets of the liver extracts identified metabolic differences between treatment groups (Supplementary Figures S1 and S2). A significant difference, was observed in the hepatic metabolic profiles measured by UPLC-MS in ESI^+^ (*P* = 0.002), although following Monte-Carlo cross-validation and false positive discovery rate corrections no significant features were positively associated with this separation (Supplementary Figure S2).

### Gall bladder tissues

No significant differences were observed between the two experimental groups in the gall bladder profiles measured by any of the analytical or statistical analyses. approaches (Figures S1 and S2).

### Biochemical summary

**Figure 3:**
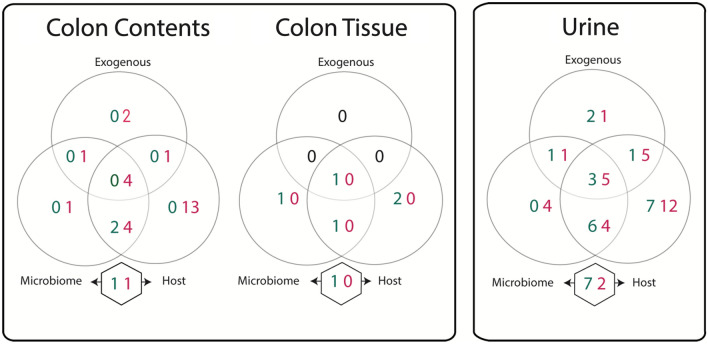
Biochemical origins of metabolites observed to differ in the colon contents, colon tissue, and urine of UNNC10201652 treated animals compared to control. Values indicate the number of metabolites increased (green) or decreased (red) with treatment and the circles indicate the source of those metabolites (i.e. if derived from the microbiome, host, or exogenously/diet). Metabolites included in the overlapping areas can be derived from either source while the metabolites listed in the hexagons are those arising from microbial-host co-metabolism. A metabolite list is provided in Supplementary Table S6.

No metabolites were found to be altered in the systemic circulation following administration of the inhibitor. Similarly, no metabolites were perturbed in the liver or gall bladder, tissues involved in the enterohepatic circulation. However, the inhibitor did modify metabolites in samples reflecting routes of excretion, including the urine, feces and colon contents. Modest differences were also noted in the colonic tissue. The absence of significant changes in the metabolic profile of the systemic circulation, but a number of changes in that of the urine probably reflects the capacity of the kidney to excrete them thereby ensuring that homeostasis was maintained. The metabolites identified in the urine, colon contents and colon tissue as altered in mice following UNC10201652 treatment were classified based on their origin as host-, microbial-, exogenously-derived, arising from microbial-host co-metabolism or the host metabolism of exogenous inputs (Fig. 3, metabolites listed in Supplementary Table S6). Several microbial-related metabolites were modulated in the colon contents and urine but negligible effects were seen in the colon wall. For example, in the colonic contents 1 metabolite of microbial origin, 4 of either host or microbial origin, 1 of either microbial or exogenous, and 4 either host, microbial or exogenous origin were reduced with treatment as well as 1 microbial-host co-metabolite while 1 microbial-host co-metabolite was increased. In the urine, 4 microbial metabolites were reduced, as were 4 microbial or host metabolites, 1 microbial or exogenous metabolite, and 5 microbial, host or exogenous metabolites. Conversely, 6 microbial or host metabolites, 1 microbial or exogenous metabolite, and 3 microbial, host or exogenous metabolites were increased with the inhitior. In addition, 7 microbial-host co-metabolites were increased and 2 were decreased. In the colon wall, treatment resulted in the increased abundance of 1 microbial, 1 microbial or host, 1 microbial, host or exogenous-derived metabolite and 1 microbial-host co-metabolite.

## Discussion

In this work the impact of an orally administered chemical inhibitor of gut bacterial β-glucuronidases was studied on the intestinal, enterohepatic, and systemic biochemisty of the mammalian supraorganism. Although inhibition of these enzymes has been previously shown to have a limited impact on the diversity and composition of the intestinal microbiota^10,15^, it was clear from this work that the functional capacity of the gut microbiota, and therefore host exposure to its biochemical output, was modified. The extent of these alterations were largely restricted to the gut with downstream changes also seen in urinary metabolic phenotypes. However, no differences were observed in the metabolic signatures of the liver, gall bladder or plasma. This suggests that the inhibitor had a minimal impact on the biochemistry of the liver or systemic circulation. As such it would appear that that the metabolic system of the host was resilient to short-term functional losses within the microbiome. While functional redundancy within the microbiota permits the loss of compositional units without disrupting its overall functional capacity, it appears that the host itself can maintain functional competency following temporal ablation of specific microbial functions. Such resilience in the overall supraorganism is essential for its fitness. These observations are in stark contrast to antibiotic exposure, which can result in the broad depletion of many microbial metabolic processes, and which has been shown to drive profound metabolic shifts throughout the host. This illustrates the potential of targeted microbial interventions as an adjunctive therapy to improve drug outcomes.

As expected, the β-glucuronidase inhibitor had a number of effects on the excretion profile of glucuronides. This included an increase in the urinary excretion of phenol glucuronide and *p*-cresyl glucuronide. However, the urinary excretion of several other glucuronides was decreased in the gut. This included glucuronides of xanthurenic acid, dihydrocaffeic acid, dihydroxyquinoline, and 2-indole-carboxylic acid. Differences in the sites of production, excretion and hydrolysis of these glucuronides may underlie these observations. Alternatively, it could be driven by the specificity of the inhibitor. UNC10201652 is restricted to “Loop 1” β-glucuronidase enzymes largely from *Firmicutes* and *Proteobacteria* but absent from *Bacteroidetes*^10,14^. There is a well-described set of distinct active site geometries for gut microbial β-glucuronidases (e.g.*,* Loop 1, Loop 2, etc*.*), and these enzyme orthologs have differential activities with distinct substrates^3,16–21^. Based on these findings, UNC10201652 appears to primarily affect β-glucuronidase orthologs that hydrolyze glucuronic acid placed on non-substituted phenols, or only para-substituted phenols, which is the case of *p-*cresyl glucuronide. Several other metabolites arising from the co-metabolism between the intestinal microbiota and the conjugative metabolism of the host were altered following exposure to the inhibitor. These included products of sulfation such as *p*-cresyl sulfate and 3-indole sulfate, both of which increased in urine, and *p*-coumaric acid sulfate and ferulic acid sulfate, which decreased. Products of *N*-acetylation, which can be performed by both the intestinal microbiota and host^22^, were also disrupted by the inhibitor, with *N*-acetyl-histidine, *N*-acetyl-glutamate, *N*-acetyl-glutamine and *N*-acetyl-lysine being increased in the urine, while *N*-acetyl-neuraminic acid and *N*-acetyl-spermidine were decreased. This indicates that the inhibitor alters metabolism beyond simply changing patterns of glucuronidation.

The abundance of serotonin in the colonic lumen was also reduced following intake of the inhibitor. It is plausible that this observation may be associated with inhibition of the bacterial hydrolysis of host-derived serotonin-glucuronide. Consistently, Walsh et al*.* have shown in vitro that diminished bacterial β-glucuronidase activity reduced serotonin concentrations^23^. Increased urinary excretion of 5-hydroxyindole acetate (5-HIAA), the primary metabolite of serotonin produced by the host, was also observed in our studies in the mice receiving β-glucuronidase inhibitor. In addition to serotonin, a number of tryptophan-related metabolites were modulated in the gut contents following inhibitor intake, including reduced amounts of prolyl-tryptophan and tryptophan-leucine, and catabolites such as indole-3-carbaldehyde, indole-acrylic acid and 3-indoxyl sulfate. Thus, further investigation of these pathways is warranted.

In the urine, several metabolites related to microbial metabolism were altered following administration of the inhibitor. These changes included metabolites involved in choline (production of TMAO from TMA), vitamin (biotin), polyamine (putrescine derivatives), and indole (indole-3-carbaldehyde, indole-acrylic acid) metabolism^24^. These observations suggest that β-glucuronidase inhibition may have a wider functional impact on the microbiome beyond glucuronidases alone. Indeed, bacterial tyrosine metabolism also appeared to be altered by UNC10201652: phenylacetylglycine was increased in the treated mice whilst tyrosine and its microbial degradation products, *p*-hydroxyphenyllactic acid and *p*-coumaric acid (as the sulfate conjugate), were decreased compared to the control animals.

The value of β-glucuronidase inhibitors in modifying the toxicity of irinotecan^8–10,25^ and a number of NSAIDS^11–13,26,27^ has already been demonstrated in animal models. The results of the present study indicate that the β-glucuronidase inhibitor UNC10201652 at low “pharmacological” doses does not result in significant changes in the metabolism of the host and the metabolic changes observed were largely confined to the gut. This provides further support for this attractive approach to limit adverse drug reactions caused by the microbiota. Furthermore, this experimental approach assessing the global metabolome after selectively targeting a specific microbial enzyme in a mouse with a full complement of microbes, offers a more natural tool to study microbial-host metabolic interactions and their biochemical reach within the context of a mammalian supraorganism. This is in contrast to the extreme germ-free and gnotobiotic models, and the variable fecal microbial transplant and antibiotic-treated models.

## Conclusion

While administration of a targeted gut bacterial β-glucuronidase inhibitor did alter the biochemical output of the microbiome and its flow to the host, these changes to the biomolecular landscape of the overall supraorganism were confined to the gut-facing aspect of the enterohepatic interface. Minimal impact was noted on the systemic metabolic system of the host at these low “therapeutic” doses. Such limited impact may be the result of the specificity of UNC10201652 to a narrow spectrum of gut bacterial β-glucuronidases. This work neatly demonstrates an experimental approach to investigate the local and systemic reach of specific bacterial enzymes and functions against the background of a fully colonized gut.

## Materials and methods

The β-glucuronidase inhibitor UNC10201652 (Inh 9, [4-(8-(piperazin-1-yl)-1,2,3,4-tetrahydro-[1,2,3]triazino[4′,5′:4,5]thieno[2,3-c]isoquinolin-5-yl)morpholine]) was synthesized at the University of Carolina at Chapel Hill as described^14^.

### Study design

All mouse experiments were performed using protocols approved by the University of North Carolina Institutional Animal Care and use Committee. The study was designed bearing in mind the Essential 10 ARRIVE guidelines and the experiments were performed in accordance to these guidelines and the corresponding regulations.

15 C57/BL6 mice were purchased from Jax (3 males aged 8.5 weeks, and 4 males and 8 females aged 10 weeks), and allowed to acclimatize to the UNC vivarium for five days prior to study start. Animals were housed in sterilized cages containing corn-cob bedding, with two enrichment items (sterile nestlets, plastic tunnels) provided within each cage. Animals had ad libitum access to standardized, irradiated mouse chow and sterile, acidified water. Pre-treatment control urine and fecal samples were collected. Eight of the mice were randomly assigned to the treatment group (2 males aged 8.5 weeks; 2 males aged 10 weeks; and 4 females aged 10 weeks) and received 20 μg of UNC10201652 by oral gavage (0 h; dissolved in DMSO at a concentration of 2 mg/mL prior to being diluted with sterile 0.9% saline and pre-warmed). Control mice (1 male aged 8.5 weeks; 2 males aged 10 weeks; and 4 females aged 10 weeks) received inhibitor-free vehicle (sterile 0.9% saline solution). A second and third dose of the inhibitor or vehicle, as appropriate, was delivered at 24 h and 48 h respectively (Table S1). Animals were housed in pairs with matching sex, age, and treatment group. The control male mouse aged 8.5 weeks was housed singly (Table S1). Urine and fecal samples were collected when possible at 24 h and 48 h after commencing the first dose before the follow-up doses were administered. Following a wash-out period of 48 h after the last treatment (96 h after commencing the first dose), mice were euthanized per the humane standards recommended by the American Veterinary Medical Association, with all measures taken to minimize pain and distress. Per these guidelines, mice were deeply anesthetized using CO_2_ until respiration was arrested. After a minimum of one minute following respiratory arrest, euthanasia was performed with a secondary method (cervical dislocation). Urine, feces, plasma, liver, colon tissues, colon contents and gall bladders were collected at necropsy (Table S1). Samples were shipped to the United Kingdom where they were prepared for ^1^H NMR spectroscopy and UPLC-MS as described below. Statistical tests to determine outliers, as well as data analysis techniques and associated statistical tests are described in subsequent sections.

### ^1^H NMR spectroscopy sample preparation

Urine samples were thawed, vortexed and centrifuged at 10,621*g* for 10 min at 4˚C before being diluted (1:1) with 30 μL urine buffer (1.5 M KH_2_PO_4_, 2 mM NaN_3_, 1% trimethylsilylpropionate (TSP) solution, 100% D_2_O, pH = 7.4) and mixed^28^. Samples were centrifuged at 20,783*g* for 10 min at room temperature and 50 μL was transferred into an 1.7 mm NMR tube. Plasma samples were thawed, vortexed and mixed in a 1:1 ratio with 100 μL of plasma buffer (0.075 M NaH_2_PO_4_, 2 mM NaN_3_, 1% TSP solution, 100% D_2_O, pH = 7.4)^28^. Samples were centrifuged for 5 min at 15,294*g* at 4 °C and 180 μL was transferred into 3 mm NMR tubes. Liver and colon tissues were thawed on ice. Approximately 20–30 mg of each sample was weighed and transferred into bead beating tubes. For extraction CHCl_3_: MeOH (2:1 V:V) was added (in a fume hood) to the tissue (300 μL) with 1 mm Zirconium beads (~ 10) before vortexing and two cycles of bead beating (40 s at 6,500 Hz speed; Percellys bead beater). H_2_O (300 μL) was added to the homogenate before centrifugation at 20,783*g* for 10 min at room temperature. The organic and aqueous layers were separated by pipette before repeating the extraction process to enhance recovery. Here, 300 μL of CHCl_3_:MeOH (2:1) and 300 μL of H_2_O was added to the remaining pellet. The mixture was vortexed, centrifuged, and the aqueous and organic phases were separated and combined with the previous phases. Organic extracts were evaporated overnight in a fume hood and aqueous extracts were dried using the Savant vacuum concentrator (180 min, 45 °C, V-AQ mode). Dried extracts were stored at − 40 °C until the day of the analysis, during which the aqueous extracts were thawed, resuspended in 700 μL of D_2_O: H_2_O (9:1 V:V, 1 mM TSP), vortexed and centrifuged at 6797*g* for 10 min at 4 °C. A volume of 600 μL was then transferred into 5 mm NMR tube. A pooled sample was prepared for each sample type for quality control (QC) purposes^29,30^ and was made by mixing 10 µL of each sample for urine and 20 µL of each sample extract for the liver, colon and plasma respectively.

### ^1^H NMR spectroscopic analysis

^1^H NMR analysis was performed on a Bruker 600 MHz spectrometer (Bruker Biospin, Karlsruhe, Germany) operating at 310 K for the plasma samples and 300 K for all other sample types. The parameters used for acquisition were as previously reported for urine and plasma^28^. Standard 1D noesy experiments were performed for all samples except plasma, incorporating a water pre-saturation step. For the plasma samples, a Carr-Purcell-Meiboom-Gill (CPMG) experiment was performed with water pre-saturation. For the tissue samples, 64 scans were acquired after 4 dummy scans for each sample, and 4 dummy scans were followed by 32 scans for the urine and plasma samples. The spectral data was imported into Matlab using in-house scripts (version R2014a, The Mathworks Inc.). The residual water region was removed (δ 4.82 ± 0.15) from all datasets and the urea (δ 5.92 ± 0.24) and dimethylsulfoxide (DMSO) signals (δ 3.18 ± 0.02 and δ 2.73 ± 0.02) were removed from the urine profiles. The urine and the liver datasets were normalized using the probabilistic quotient method before manual alignment of the spectral data. The colon tissues and plasma datasets were not normalized. Principal components analysis (PCA) was performed for each dataset to identify any outliers. For the urine dataset, mean-centering and Pareto scaling was applied. For the plasma, liver and colon tissues, the datasets were mean centered only. One plasma spectrum from a control mouse was identified as an outlier due to difficulties aligning it to the other spectra and was excluded from future analysis. Metabolite annotation was performed by comparison to an in-house database.

### UPLC-MS sample preparation

The urine samples were prepared according to Gray et al.^31^. Briefly, samples were thawed and vortexed, before being combined with ice cold MeOH (1:3 V:V), vortexed and stored overnight at − 20 °C. A ten minute centrifugation at 4 °C with 14,000*g* was performed before a 1:10 dilution of the supernatant with H_2_O in 96 well-plates, which was centrifuged immediately prior to analysis at 700*g* for 5 min. Samples were analyzed in a randomized order. If available, a biological duplicate was prepared, this was particularly valuable for liver samples where a large amount of heterogeneity existed across the tissue.

Liver samples were prepared according to Want et al.^32^. Briefly, samples were thawed and a first replicate of 50 mg (± 0.30 mg) was collected followed by a second replicate of 50 mg (± 0.30 mg). Zirconium beads (~ 100 μL) and 1.2 mL of cold MeOH:H_2_O (1:1 V:V) were added to each replicate before homogenization with two cycles of Percellys bead beater (40 s, 6500 Hz), centrifugation at 10,000*g* (10 min, 4 °C) and collection of the supernatant (250 μL). All samples were concentrated overnight under nitrogen flow at room temperature and the dried extracts were stored at − 40 °C until the day of analysis. The same protocol was used for the preparation of colon tissue, with the exception of a volume of 1.5 mL of cold MeOH: H_2_O (1:1 V:V), instead of 1.2 mL, to 48.93 ± 3.18 mg of tissue. Also, the limited quantity of the samples available did not allow the preparation of biological replicates, but technical replicates were analyzed. Gall bladders were collected and frozen intact. Metabolite extraction from the gall bladders was performed on the entire organ and contents, which presented small and variable weights (1.93 ± 2.26 mg). To achieve this, the volume of cold MeOH: H_2_O (1:1 V:V) was adjusted for each sample to maintain the ratio of 0.8 mg tissue: 200 μL of cold MeOH:H_2_O. The rest of the protocol was the same as for the liver or colon tissues metabolite extraction. The liver and colon tissue extracts were resuspended in 120 μL of MeOH:H_2_O (1:1 V:V), while the gall bladders were resuspended into 43.5 μL, before to being vortexed, centrifuged (5 min, 20,783*g*, room temperature), randomized and analyzed.

Feces and colon contents were prepared using a similar method to the liver tissues. However, H_2_O was used for the metabolite extraction step with 3.3 mM NaN_3_ to prevent bacterial growth, and the weight of the samples was variable with some providing less than 10 mg of material. The volume of the solvent used was by consequence adjusted to the weight of each sample maintaining a ratio of 50 mg sample:1.1 mL of solvent. Homogenization was performed with two cycles of Percellys bead beater (40 s, 6,500 Hz), followed by centrifugation at 13,000*g* (20 min, 4 °C). Supernatants were collected and the bead beating step was repeated with another volume of H_2_O (3.3 mM NaN_3_) to optimize metabolite recovery. After centrifugation, both supernatants were combined and stored at − 40 °C until the day of the analysis. Prior to analysis, samples were concentrated under nitrogen flow overnight, and re-suspended in 529 µL of MeOH:H_2_O (1:1 V:V) if the initial sample weight was 50 mg (or adapted if the initial sample weight was lower), vortexed, centrifuged (5 min, 20,783*g*, room temperature) and analyzed.

A pooled sample was prepared for each matrix for quality control (QC) purposes^29,30^ and was made by mixing 20 µL of each sample extracts. Sample randomization was applied for each matrix.

### UPLC-MS analysis

Analysis was performed on a Waters Acquity I-class UPLC system (Waters Corp., Milford, MA) and separation on an HSS T3 1.8 µM column (2.1 mm i.d. × 150 mm). The temperature of the column was set at 45 °C with the autosampler temperature at 4 °C. The volume of sample injected was 1 µL. The mobile phase used was water containing 0.1% (V:V) formic acid (solvent A) and ACN containing 0.1% (V:V) formic acid (solvent B). The initial mobile phase composition was composed 99% solvent A with a flow rate of 0.6 mL/min. The proportion of solvent B was set to increase from 1% at 0.10 min to reach 55% at 10 min. The percentage of solvent B was then gradually increased in 0.15 min intervals of 10% before 100% was reached at 10.70 min. This solvent composition was maintained for 0.95 min as a wash step followed by a re-equilibration step of 2 min with 99% of solvent A (total run time 12.65 min). Volumes of 1000 μL of weak wash solvent (H_2_O:ACN 3:1 V:V) and of 1000 μL of strong wash solvent (IPA) were used. Fifteen of the pooled QC samples were used at the beginning of the analysis of each matrix to condition the column^29,30^ and a QC was injected every 5 samples throughout the analysis to monitor its repeatability. The Acquity UPLC-MS system was interfaced with the Waters Xevo G2 Q-ToF mass spectrometer (Waters Corp., Wilmslow, U.K). In positive electrospray ionisation (ESI^+^), the capillary voltage was 1.5 kV, the source temperature was set at 120 °C, the cone gas flow was 50 L/h, the desolvation gas temperature was 600 °C and the desolvation gas flow was 1000 L/h. The same parameters were applied to negative electrospray ionisation (ESI^−^), apart from the capillary voltage which was 1 kV, and the cone gas flow which was 100 L/Hr. The acquisition was carried out over the *m*/*z* range 50–1200. Centroid mode with MS^e^ acquisition was applied to the experiment with a RAMP collision energy going from 15 to 45 eV. Leucine encephalin (MW = 556.27 Da) was infused for mass accuracy at a flow rate of 10 μL/mL with scan acquisition every 60 s. The data were collected using MassLynx V 4.1 (Waters Corp., Manchester, U.K.).

The raw datafiles were imported into R and converted into .mZML format using the MSConvert utility of the ProteoWizard package 3.0^33^. The pre-processing of the data was performed with the XCMS package^34–36^. Peak picking was performed using the centWave method (parameters adapted according raw data exploration). Peak grouping was performed using the nearest method. Normalization of the datasets was performed using median fold change normalization and filtered based on the coefficient of variation of each variable, using a threshold of 20% compared to the quality control pooled sample. Mean-centering and log-transformation, with an offset of 20, was applied.

Unsupervised statistical analyses were performed using SIMCA 14.1 (Umetrics, Umea, Sweden) to assess the presence of outliers. The supervised approach, covariate-adjusted projection to latent structures discriminant analysis (CA-PLS-DA^37^) was performed in Matlab R2018a. Supervised models were adjusted for any bias that could have been introduced due to batch, sex, age, cage or biological/technical replicate cofounders. The goodness of fit and the predictive power of each PLS-DA model was assessed by studying the R^2^ and Q^2^ values, respectively. Wilcoxon rank sum test was used to calculate a *p* value and to estimate the significance of the separation along the unique component of the models. If the *p* value was significant (α < 0.05), features identified in the skyline significance plot as having a Q-value (*p* value corrected by a multiple testing based on the Benjamini–Hochberg method^38^ with a false discovery rate of 5%) after a Monte-Carlo Cross-Validation (MCCV, using 100 rounds and a partitioning of 5) below 0.05, were selected for metabolite identification. Features found to significantly influence the model were putatively annotated using different approaches. Initially, a correlation script was used to highlight features derived from the same metabolite (isotopes, adducts, dimers). To aid putative identification features were also matched with the *m*/*z* and the retention times of the compounds in the National Phenome Centre (NPC) in-house database. In addition, features were also matched with online databases (HMDB, Metlin and LipidMaps) using CEU Mass Mediator^39^, by comparing the [M+H]^+^, [M+H-H_2_O]^+^, [M+Na]^+^ or [M+K]^+^ adducts for ESI^+^ datasets and [M-H]^−^ and [M+Cl]^−^ and [M-H-H_2_O]^−^ for ESI^−^ datasets, as well as the main fragments. The level of confidence for each annotation are reported according the criteria used by the Metabolomics Society^40^, with the addition of a sub-confidence group in the level 2 annotation. Annotation to a specific metabolite done on one orthogonal parameter (e.g., *m*/*z* values matching to database) or two orthogonal parameters (e.g., *m*/*z* values and retention time) without spiking the corresponding authentic standard are reported as annotation level 2b and 2a, respectively. The − log(q value) of the metabolites successfully annotated by UPLC-MS were used to generate heatmaps to visually assess their variation in the mice group treated with the inhibitor compared to the control mice group.

## Supplementary Information

Supplementary Information.

## Data Availability

The data will be made available upon acceptance for publication. The datasets generated and analysed during the current study are available from the first authors on reasonable request.
