# Supplementary material for: Characterizing the metabolic effects of the selective inhibition of gut microbial β-glucuronidases in mice
Source: Sci Rep. 2022 Oct 19;12:17435. doi: 10.1038/s41598-022-21518-4 (PMC9581996; doi:10.1038/s41598-022-21518-4)
Supplement: Supplementary file 1 — Supplementary Information. [file 41598_2022_21518_MOESM1_ESM.docx]

**Supplementary Information for the Manuscript:**

**Characterizing the metabolic effects of the selective inhibition of gut microbial β-glucuronidases in mice**

Marine P. M. Letertre^1,2^, Aadra P. Bhatt^3^, Michael Harvey^4^, Jeremy K. Nicholson^5,6^, Ian D. Wilson^1^, Matthew R. Redinbo^7^, Jonathan R. Swann^1,4^*

1. Department of Metabolism, Digestion and Reproduction, Imperial College London, UK.
2. Nantes Université, CNRS, CEISAM, UMR 6230, F-44000 Nantes, France.
3. Department of Medicine, University of North Carolina, Chapel Hill, NC 27599, USA.
4. School of Human Development and Health, Faculty of Medicine, University of Southampton, UK.
5. The Australian National Phenome Centre, Health Futures Institute, Murdoch University, Perth, AU.
6. Institute of Global Health Innovation, Faculty of Medicine, Imperial College London, London, UK.
7. Departments of Chemistry, Biocemistry, Microbiology and Genomics, University of North Carolina, Chapel Hill, NC 27599, USA

**
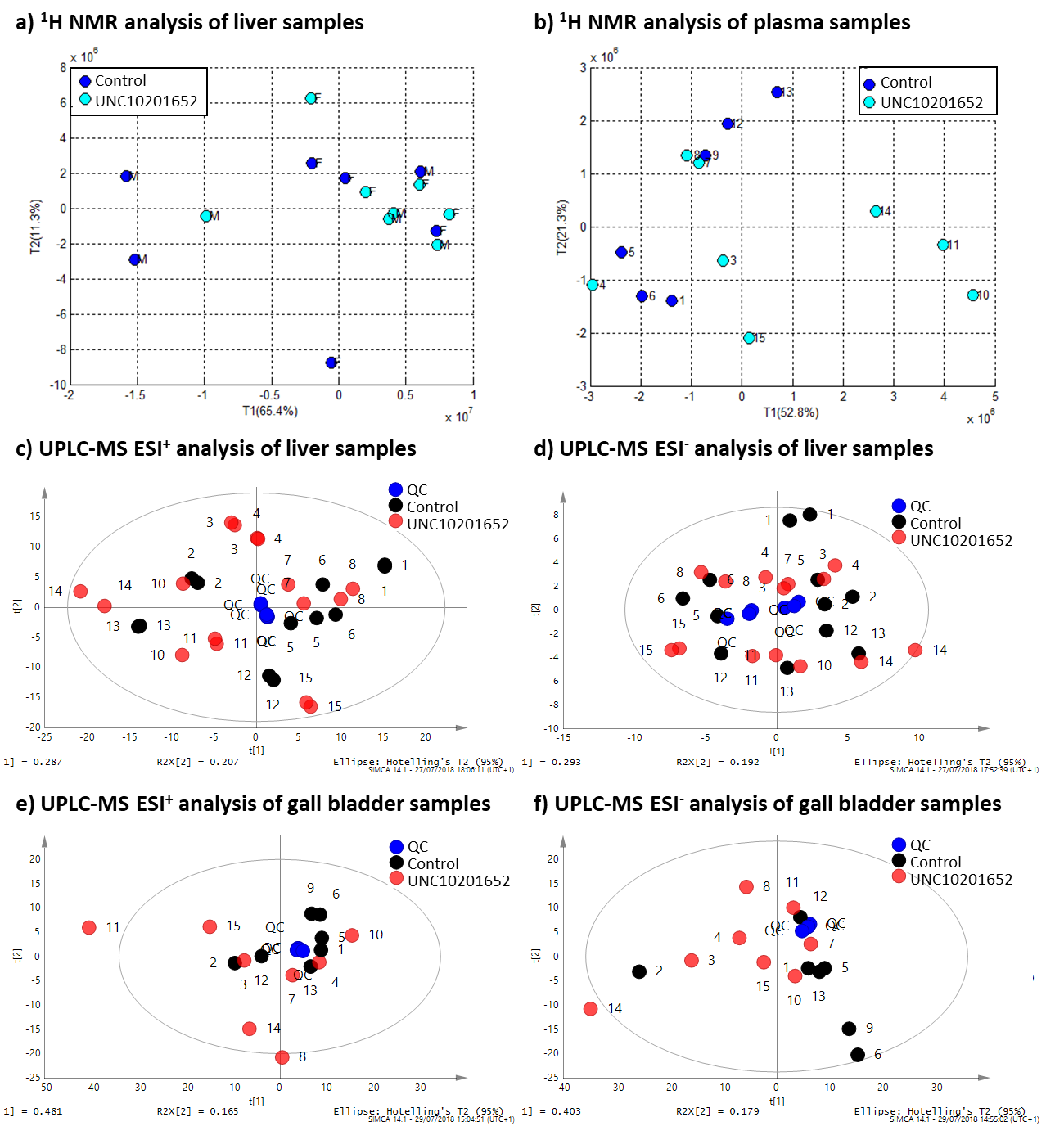
**

**Supplementary Figure S1. PCA scores plots obtained for the ^1^H NMR spectroscopy datasets of the liver extracts and plasma samples, for the UPLC-MS ESI^+^ and the UPLC-MS ESI^-^ datasets of the liver extracts and for the UPLC-MS ESI^+^ and the UPLC-MS ESI^-^ datasets of the gall bladders.** Each point represents an individual sample. All samples reported in this figure were collected only after animal euthanasia, the time point considered is thus 96 h following the first dose of the inhibitor. Biological replicates were used for UPLC-MS analysis of the liver samples.


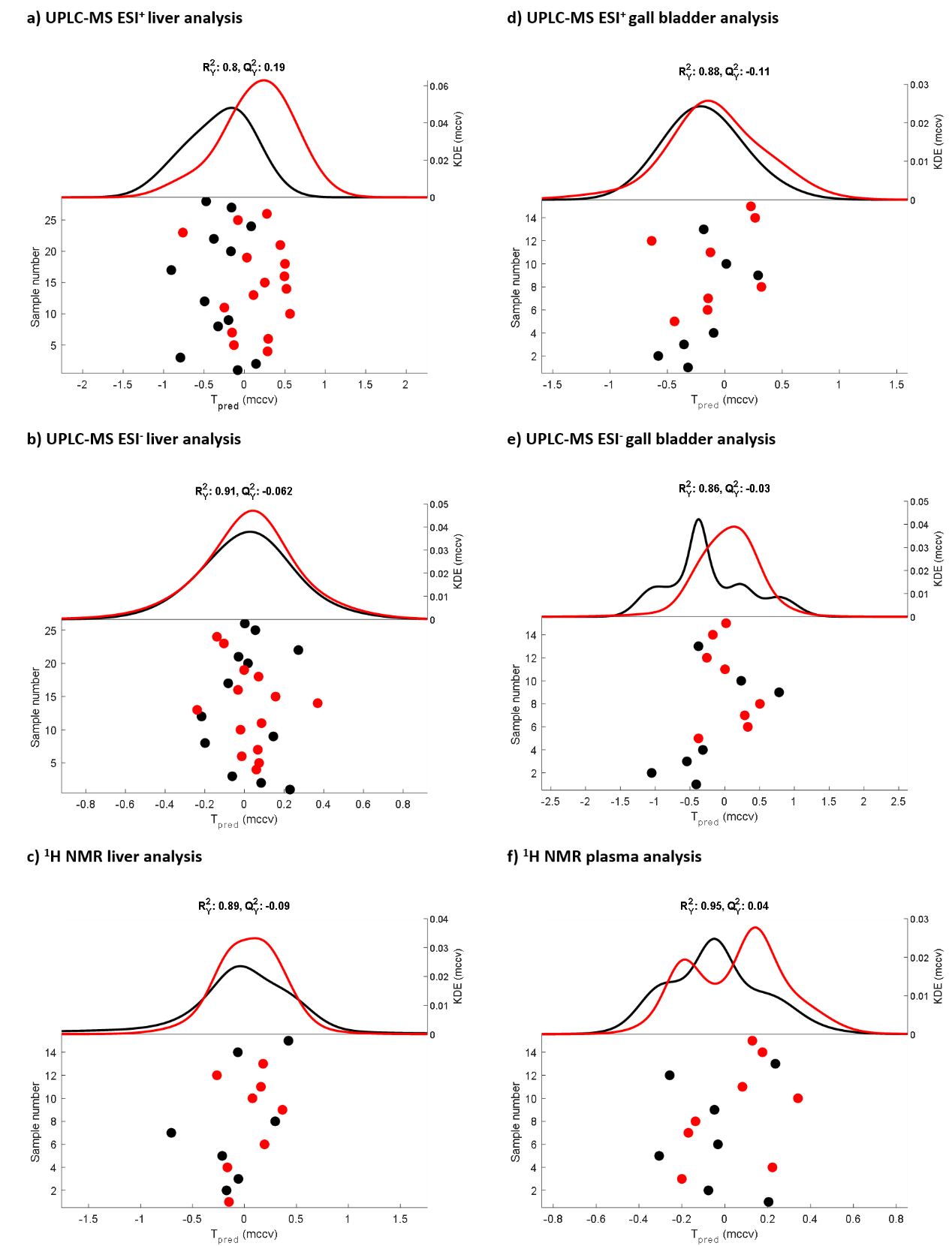


**Supplementary Figure S2. CA-PLS-DA analysis comparing the plasma, liver, and gall bladder metabolic profiles between control and treated animals.** The CA-PLS-DA scores plots and associated kernel density estimation (KDE) are shown for the models comparing the hepatic biochemical profiles of control (black) and treated (red) animals measured by (a) UPLC-MS ESI^+^ (*P* = 0.001), (b) UPLC-MS ESI^-^ (*P* = 0.938) and (c) ^1^H NMR spectroscopy (*P* = 0.613). CA-PLS-DA models comparing the gall bladder profiles measured by (d) UPLC-MS ESI^+^ (*P* = 0.694) and (e) UPLC-MS ESI^-^ (*P* = 0.463) and the plasma profiles measured by (f) ^1^H NMR spectroscopy (*P* = 0.535) are also shown. Each point represents an individual sample. All samples reported in this figure were collected only after animal euthanasia, the time point considered is thus 96 h following the first dose of the inhibitor. Biological replicates were used for UPLC-MS analysis of the liver samples, and this was adjusted for in the CA-PLS-DA model.


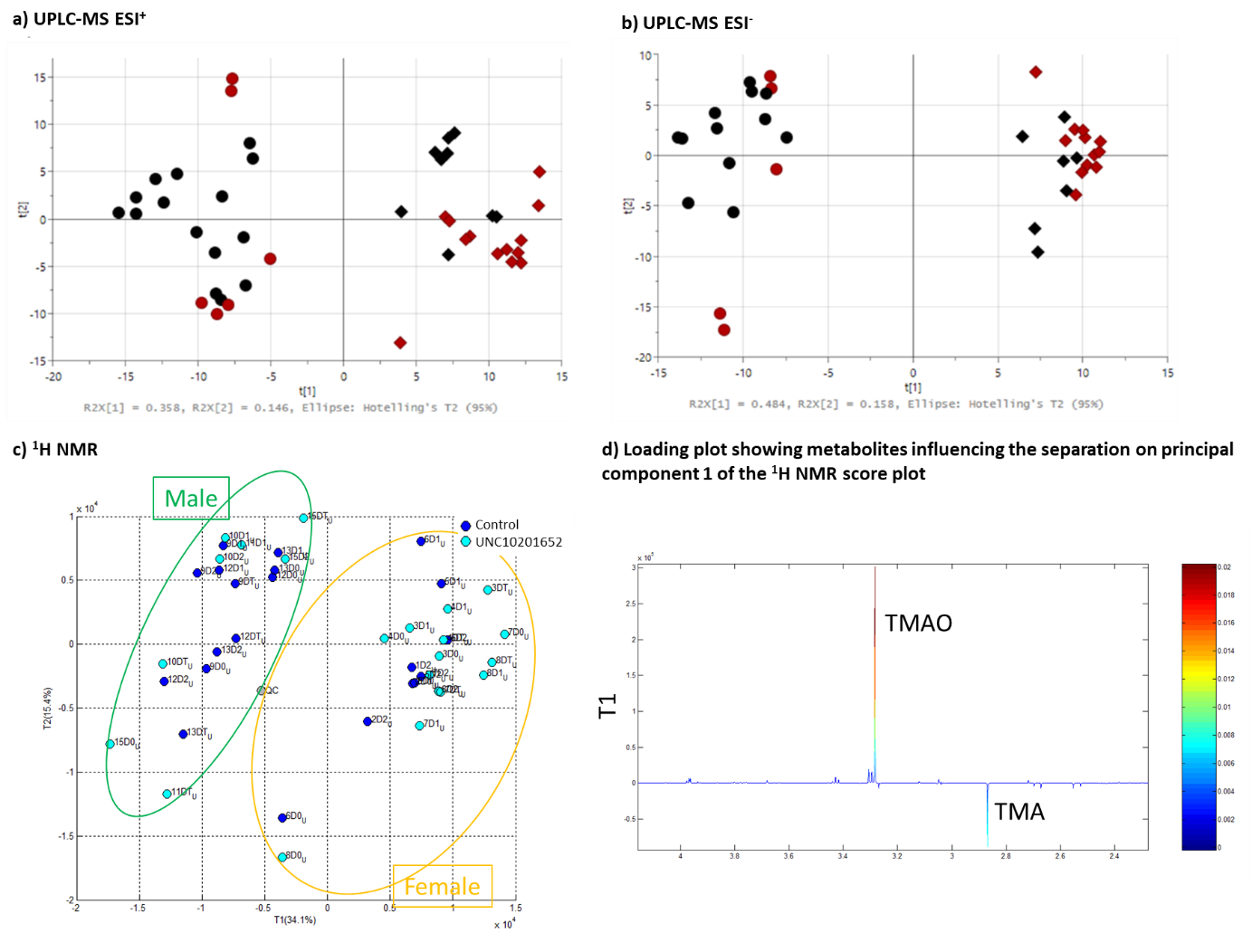


**Supplementary Figure S3. Unsupervised analysis performed on the UPLC-MS and ^1^H NMR spectroscopic urine sample dataset.** PCA scores plots obtained by both UPLC-MS ESI^+^ and UPLC-MS ESI^-^ of the urine samples, where male samples are displayed with circle symbols and female samples are displayed with diamonds. PCA scores plot obtained by ^1^H NMR spectroscopy for the urine samples and the loading plot and showing the metabolites driving the separation along principal component 1. The loading plots linked to the NMR PCA showed that the separation between male and female mice was due to higher concentrations of TMAO in females and of TMA in males. Each point represents an individual sample. All the samples available at the different post-dose time points (24, 48 and 96 h following the first dose of UNC10201652) have been considered. Technical replicates were included when sufficient material was available.


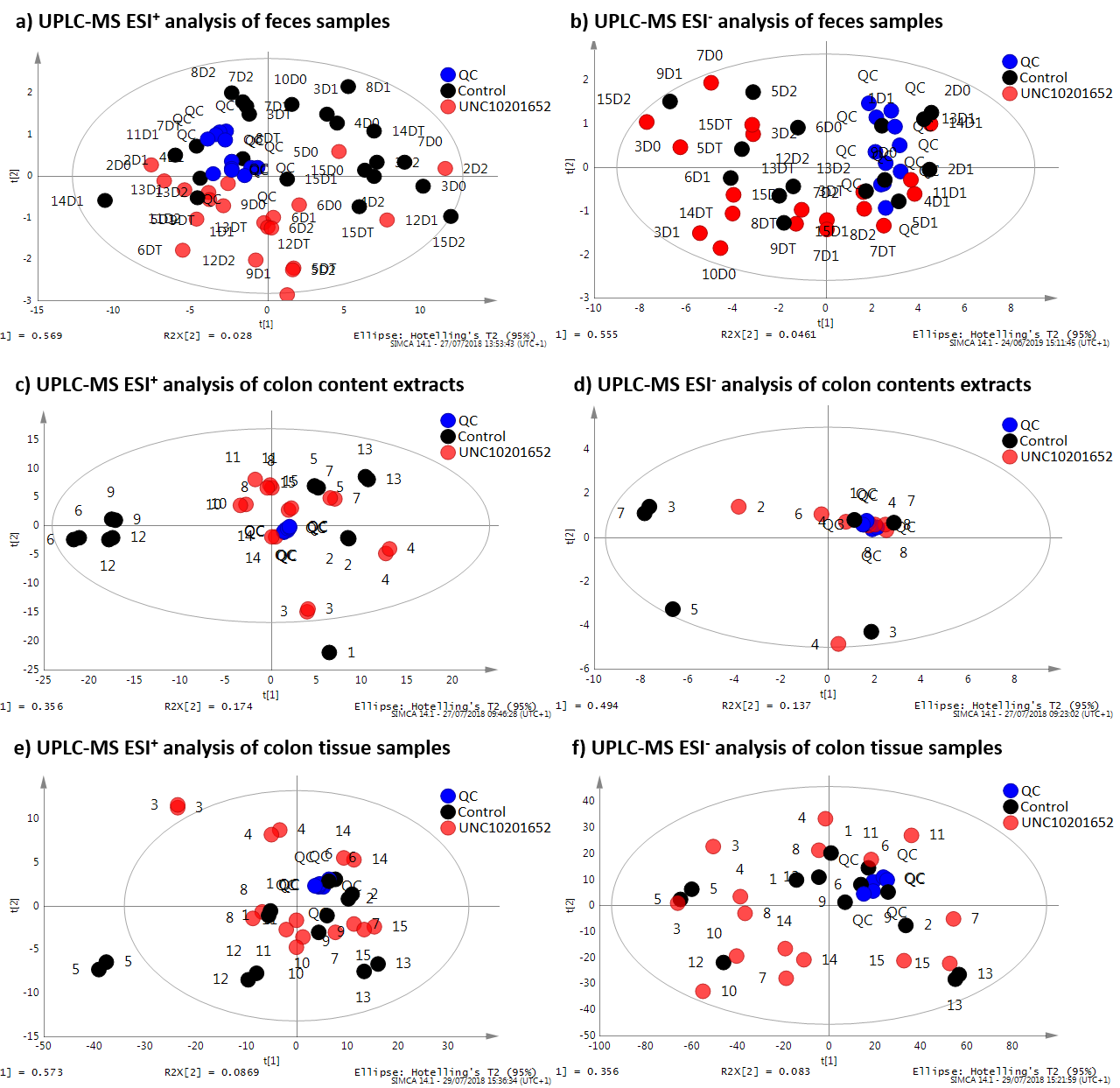


**Supplementary Figure S4. Unsupervised analysis performed on the UPLC-MS and ^1^H NMR spectroscopic profiles obtained from the feces, colon contents and colon tissue extracts.** PCA scores plots obtained from the profiles of the feces, colon contents, and colon tissue samples measured by NMR, UPLC-MS ESI^+^ and UPLC-MS ESI^-^. Control samples are shown in black, Inhibitor treated samples in red and C samples in blue. Each point represents an individual sample. For the fecal samples, all the samples available at the different post-dose time points (24, 48 and 96 h following the first dose of UNC10201652) have been considered. For the colon contents and tissue samples, technical replicates were included when sufficient material was available.


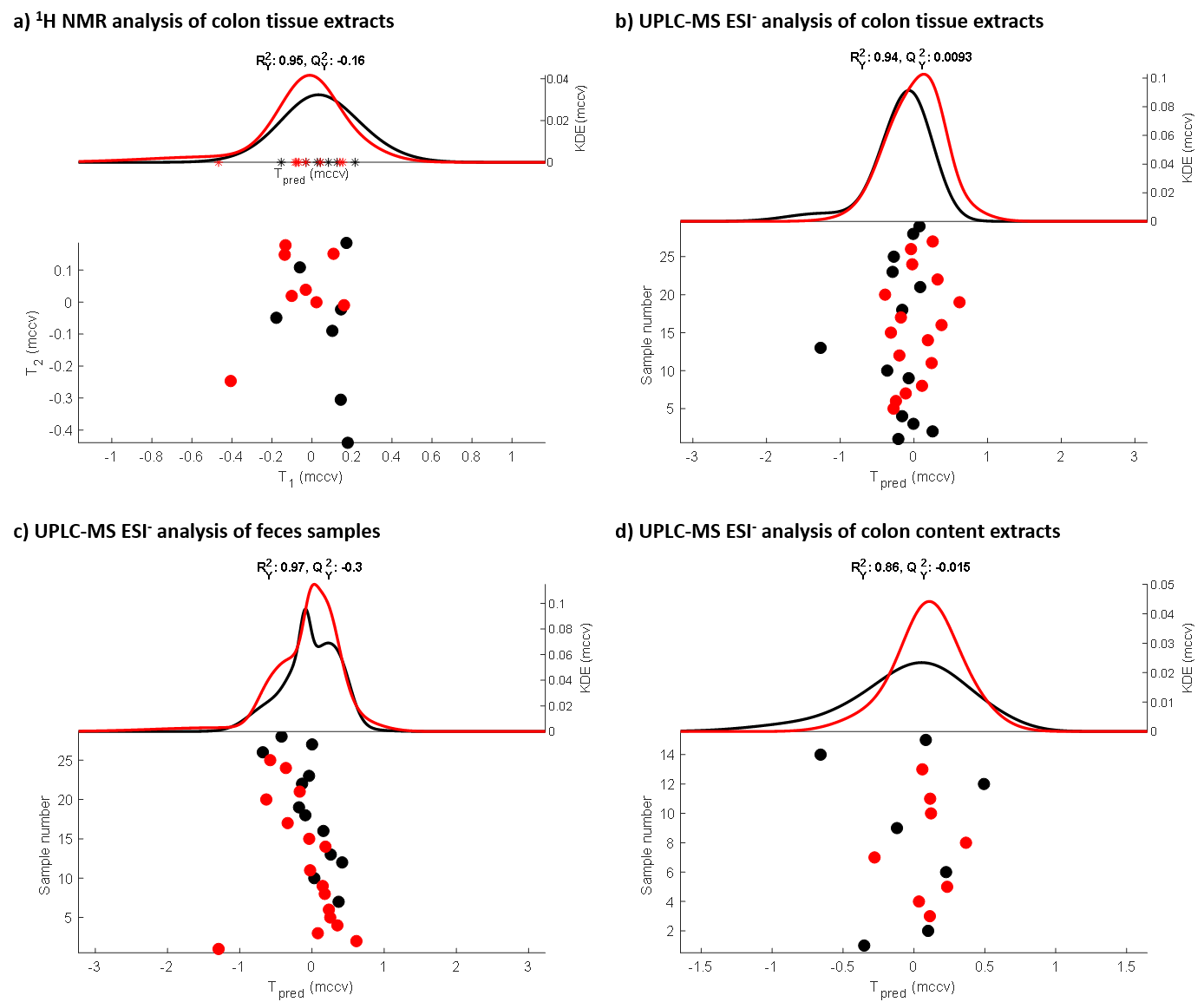


**Supplementary Figure S5. CA-PLS-DA analysis performed on the metabolic profiles of feces and colon tissues by NMR spectroscopy and UPLC-MS.** CA-PLS-DA scores plots are shown with the accompanying kernel density estimation (KDE) comparing the control (black) and treated (red) samples for colon tissue extracts measured by (a) NMR spectroscopy and (b) UPLC-MS ESI^-^and (d) UPLC-MS ESI^+^ and (c) fecal samples measured by UPLC-MS ESI^-^. Each point represents an individual sample. For the fecal samples, all the samples available at the different post-dose time points (24, 48 and 96 h following the first dose of UNC10201652) have been considered. For the colon tissue, technical replicates were included when sufficient material was available, and this information was used as a covariate in the CA-PLS-DA model.

**Table S1.** Metadata and samples collected.

|  |  |  |  |  | Baseline | | 24 post treatment D1 | | 48h post treatment D2 | | Terminal DT | | | | | | |
| --- | --- | --- | --- | --- | --- | --- | --- | --- | --- | --- | --- | --- | --- | --- | --- | --- | --- |
| Cage # | Mouse # | Treatment | Sex | DOB (MM/DD/YY) | Urine (µL) | Fecal | Urine (µL) | Fecal | Urine (µL) | Fecal | Urine (µL) | Fecal | Colon contents | Colon | Plasma (µL- h indicates hemolysis) | Gall bladder | Liver |
| 1 | ф | Saline | F | 9/27/2016 | 10 | ⌧ | ⌧ | ✓ | 20 | ✓ | ⌧ | ⌧ | ✓ | ✓ | 400 (h) | ✓ | ✓ |
| 1 | L | Saline | F | 9/27/2016 | 150 | ✓ | ⌧ | ✓ | 400 | ✓ | 10 | ⌧ | ✓ | ✓ | ⌧ | ✓ | ✓ |
| 2 | ф | UNC10201652 | F | 9/27/2016 | 100 | ✓ | 200 | ✓ | ⌧ | ✓ | 100 | ✓ | ✓ | ✓ | 500 (h) | ✓ | ✓ |
| 2 | L | UNC10201652 | F | 9/27/2016 | 20 | ✓ | 150 | ✓ | ⌧ | ✓ | 200 | ✓ | ✓ | ✓ | 500 (h) | ✓ | ✓ |
| 3 | ф | Saline | F | 10/05/2016 | 100 | ✓ | 200 | ✓ | 300 | ✓ | ⌧ | ✓ | ✓ | ✓ | 200 | ✓ | ✓ |
| 3 | L | Saline | F | 10/05/2016 | 15 | ✓ | 200 | ✓ | 300 | ✓ | 10 | ✓ | ✓ | ✓ | 300 | ✓ | ✓ |
| 4 | ф | UNC10201652 | F | 10/05/2016 | 100 | ✓ | 200 | ✓ | 300 | ✓ | 500 | ✓ | ✓ | ✓ | 200 | ✓ | ✓ |
| 4 | L | UNC10201652 | F | 10/05/2016 | 15 | ✓ | 200 | ✓ | 500 | ✓ | 500 | ✓ | ✓ | ✓ | 250 (h) | ✓ | ✓ |
| 5 | ф | Saline | M | 10/22/2016 | 500 | ✓ | 500 | ✓ | 500 | ✓ | 600 | ✓ | ✓ | ✓ | 300 | ✓ | ✓ |
| 6 | ф | UNC10201652 | M | 10/22/2016 | ⌧ | ✓ | 200 | ✓ | 150 | ✓ | 20 | ✓ | ✓ | ✓ | 200 | ✓ | ✓ |
| 6 | L | UNC10201652 | M | 10/22/2016 | ⌧ | ✓ | 100 | ✓ | ⌧ | ✓ | 150 | ✓ | ✓ | ✓ | 500 | ✓ | ✓ |
| 7 | ф | Saline | M | 10/05/2016 | 200 | ⌧ | 100 | ✓ | 20 | ✓ | 500 | ✓ | ✓ | ✓ | 200 | ✓ | ✓ |
| 7 | L | Saline | M | 10/05/2016 | 300 | ✓ | 600 | ✓ | ⌧ | ✓ | 10 | ✓ | ✓ | ✓ | 150 (h) | ✓ | ✓ |
| 8 | ф | UNC10201652 | M | 10/05/2016 | 10 | ✓ | ⌧ | ✓ | 10 | ✓ | 10 | ✓ | ✓ | ✓ | 200 | ✓ | ✓ |
| 8 | L | UNC10201652 | M | 10/05/2016 | 20 | ✓ | 20 | ✓ | 300 | ✓ | 300 | ✓ | ✓ | ✓ | 200 (h) | ✓ | ✓ |

**Table S2. Metabolite annotations putatively performed on the features found to be significantly affected by the inhibition of the bacterial β-glucuronidase in urine samples in UPLC-MS ESI+ mode.** All the m/z reported correspond to [M+H]^+^ adducts and the level of annotations are reported according the levels of confidence described in the experimental section.

| **putative ID** | **Adduct** | **formula** | **ID level** | ***m/z*** | **Calculated mass** | **frag 1 theo** | **frag 2 theo** | **frag 3 theo** | **frag 4 theo** | **frag 1 obs** | **frag 2 obs** | **frag 3 obs** | **frag 4 obs** | **RT (mins)** | **DB** | **In silico / exp** | **NPC DB** |
| --- | --- | --- | --- | --- | --- | --- | --- | --- | --- | --- | --- | --- | --- | --- | --- | --- | --- |
|  |  |  |  |  |  |  |  |  |  |  |  |  |  |  |  |  |  |
| *S*-adenosylmethionine | [M+H]^+^ | C_15_H_22_N_6_O_5_S | 2a | / | 399.1434 | 298.094 | 264.0908 | 250.0918 | 136.0604 | 298.0981 | / | 250.0933 | 136.0638 | 0.55 | Metlin 3289 | Exp | Yes |
| *N*-acetylspermidine | [M+H]^+^ | C_9_H_21_N_3_O | 2b | 188.176 | 188.1755 | 171.1488 | 117.1018 | 100.0758 | 72.0812 | 171.1496 | 117.1032 | 100.0769 | 72.0823 | 0.55 | Metlin 3323 | Exp | No |
| *N6,N6,N6*-trimethyl-L-lysine | [M+H]^+^ | C_9_H_20_N_2_O_2_ | 2a | 189.1603 | 189.1598 | 172.1332 | 171.1492 | 143.1543 | 114.1277 | / | 171.1496 | / | / | 0.55 | HMDB0001325 | Theo | Yes |
| Lysine | [M+H]^+^ | C_6_H_14_N_2_O_2_ | 2a | 147.113 | 147.1125 | 130.0857 | 106.0021 | 84.081 | 64.0165 | 130.0876 |  | 84.0818 | / | 0.55 | Metlin 25 | Exp | Yes |
| 5-hydroxylysine | [M+H]^+^ | C_6_H_14_N_2_O_3_ | 2a | / | 163.1083 | 145.0977 | 128.0712 | 117.1028 | 99.0922 | / | 128.0723 | 117.1044 | 99.0896 | 0.55 | HMDB0000450 | Exp | Yes |
| Histidine | [M+H]^+^ | C_6_H_9_N_3_O_2_ | 2b | 156.0779 | 156.0764 | 110.0713 | 95.0606 | 93.0449 | 83.061 | 110.0746 | 95.0602 | 93.0449 | 83.0622 | 0.55 | Metlin 21 | Exp | No |
| Taurine | [M+H]^+^ | C_2_H_7_NO_3_S | 2a | 126.0232 | 126.0214 | 108.0107 | 84.9596 | 78.9847 | 64.9698 | 108.012 | / | 78.9833 | / | 0.56 | Metlin 31 | Exp | Yes |
| Glutamate | [M+H]^+^ | C_5_H_9_NO_4_ | 2a | 148.0621 | 148.0607 | 130.0501 | 102.0548 | 84.0446 | / | 130.0508 | 102.0554 | 84.0448 | / | 0.59 | Metlin 19 | Exp | Yes |
| 5-aminopentanoic acid | [M+H]^+^ | C_5_H_11_NO_2_ | 2a | 118.0864 | 118.0864 | 101.0602 | 100.076 | 82.0657 | 56.0504 | / | 100.0768 | 82.0671 | 56.0501 | 0.6 | Metlin 6902 | Exp | Yes |
| Carnitine | [M+H]^+^ | C_7_H_15_NO_3_ | 2a | 162.1127 | 162.1107 | 103.0385 | 102.0908 | 82.0282 | 60.0812 | 103.0422 | 102.0933 | / | / | 0.6 | Metlin 52 | Exp | Yes |
| Thiamine | [M+H]^+^ | C_12_H_16_N_4_OS | 2a | 265.1123 | 265.1117 | 144.0476 | 122.0714 | 81.0449 | / | 144.0495 | 122.0719 | 81.0492 | / | 0.62 | Metlin 229 | Exp | Yes |
| Creatinine | [M+H]^+^ | C_4_H_7_N_3_O | 2a | 114.0671 | 114.0687 | 86.0741 | 72.0474 | / | / | 86.0722 | 72.0446 | / | / | 0.63 | Metlin 8 | Exp | Yes |
| Sucrose | [M+Na]^+^ | C_12_H_22_O_11_ | 2a | 365.1058 | 365.1051 | 203.0514 | 185.0414 | / | / | 203.0556 | / | / | / | 0.63 | Metlin 137 | Exp | Yes |
| *N*-acetylneuraminic acid | [M+H]^+^ | C_11_H_19_NO_9_ | 2a | 310.1105 | 310.1123 | 292.101 | 274.0929 | 256.0852 | 250.0927 | 292.1043 | 274.0932 | / | 250.0893 | 0.64 | Metlin 5739 | Exp | Yes |
| Trigonelline | [M+H]^+^ | C_7_H_7_NO_2_ | 2a | 138.0557 | 138.0553 | 94.0663 | 92.0506 | 78.0349 | 67.0552 | 94.0666 | 92.0506 | / | 67.0545 | 0.65 | Metlin 273 | Exp | Yes |
| Creatine | [M+H]^+^ | C_4_H_9_N_3_O_2_ | 2b | 132.0779 | 132.0768 | 115.05 | 114.0661 | 90.0555 | 87.0557 | 115.0516 | 114.0669 | 90.0555 | 87.0555 | 0.65 | Metlin 7 | Exp | No |
| *NG,NG*-dimethyl-arginine | [M+H]^+^ | C_8_H_18_N_4_O_2_ | 2b | 203.1508 | 203.1497 | 158.1247 | 133.0964 | 116.0704 | 88.0875 | 158.1294 | 133.0965 | 116.0713 | 88.0879 | 0.67 | Metlin 6891 | Exp | No |
| Glycyl-aspartate | [M+H]^+^ | C_6_H_10_N_2_O_5_ | 2a | 191.0682 | 191.0662 | 173.0557 | 155.0451 | 145.0608 | 127.0502 | 173.0563 | 155.0456 | / | 127.0471 | 0.68 | HMDB0028837 | Theo | Yes |
| *N*-acetylhistidine | [M+H]^+^ | C_8_H_11_N_3_O_3_ | 2b | 198.0873 | 198.0865 | 180.0757 | 156.0759 | 152.081 | 110.0709 | 180.0764 | 156.0771 | 152.0768 | 110.0723 | 0.71 | Metlin 44784 | Exp | No |
| Citrulline | [M+H]^+^ | C_6_H_13_N_3_O_3_ | 2a | 176.1031 | 176.1019 | 159.0757 | 116.0715 | 115.087 | 113.0706 | 159.0758 | 116.0704 | / | 113.0721 | 0.71 | Metlin 16 | Exp | Yes |
| *N*-acetylglutamine | [M+H]^+^ | C_7_H_12_N_2_O_4_ | 2a | 189.0867 | 189.0869 | 172.0603 | 147.0747 | 130.0496 | 84.0444 | 172.0616 | / | 130.0512 | 84.0455 | 0.82 | Metlin 58344 | Exp | Yes |
| 4-guanidino butanoic acid | [M+H]^+^ | C_5_H_11_N_3_O_2_ | 2a | 146.0934 | 146.0917 | 128.0817 | 104.0707 | 87.0447 | 86.0607 | 128.0822 | 104.0714 | 87.0448 | 86.0613 | 0.82 | Metlin 4155 | Exp | Yes |
| Glycyl-proline | [M+H]^+^ | C_7_H_12_N_2_O_3_ | 2a | 173.0937 | 173.0915 | 155.081 | 127.0863 | 116.0706 | 70.0655 | 155.0821 | 127.0879 | 116.0711 | 70.0663 | 0.82 | Metlin 4185 | Exp | Yes |
| *N-*acetyllysine | [M+H]^+^ | C_8_H_16_N_2_O_3_ | 2a | 189.1242 | 189.123 | 172.0961 | 171.1124 | 147.1123 | 129.102 | 172.0972 | 171.1133 | 147.112 | 129.1016 | 0.83 | Metlin 63102 | Exp | Yes |
| *N-*acetylputrescine | [M+H]^+^ | C_6_H_14_N_2_O | 2a | 131.0901 | 131.184 | 114.0915 | 72.0816 | / | / | 114.0922 | 72.0818 | / | / | 0.83 | HMDB002064 | Exp | Yes |
| *N-*acetylglutamic acid | [M+H]^+^ | C_7_H_11_NO_5_ | 2a | 190.0736 | 190.076 | 172.0611 | 148.0601 | 130.0495 | 102.0559 | 172.063 | 148.06 | 130.0513 | 102.0544 | 1.35 | Metlin 3325 | Exp | Yes |
| Uridine | [M+H]^+^ | C_9_H_12_N_2_O_6_ | 2a | / | 133.0489 | 115.0372 | 113.0349 | 97.0298 | 85.0289 | / | 113.0365 | / | / | 1.38 | Metlin 90 | Exp | Yes |
| Tyrosine | [M+H]^+^ | C_9_H_11_NO_3_ | 2a | 182.0849 | 182.0864 | 165.0548 | 147.0451 | 136.0753 | 123.0424 | 165.0538 | 147.043 | 136.0755 | 123.0423 | 1.43 | Metlin 34 | Exp | Yes |
| 3-hydroxyanthranilic acid | [M+H]^+^ | C_7_H_7_NO_3_ | 2b | 154.0497 | 154.049 | 136.0386 | 108.0441 | 80.0498 | / | 136.0399 | 108.0437 | 80.0501 | / | 1.47 | Metlin 3275 | Exp | No |
| Leucine | [M+H]^+^ | C_6_H_13_NO_2_ | 2a | 132.1011 | 132.1016 | 86.0969 | 73.067 | 69.07 | / | 86.0973 | / | / | / | 1.56 | Metlin 24 | Exp | Yes |
| 4-pyridoxate | [M+H]^+^ | C_8_H_9_NO_4_ | 2a | 184.0603 | 184.0604 | 166.0495 | 148.0387 | 144.9871 | 138.0558 | 166.0508 | 148.0401 | 144.9589 | 138.0536 | 1.56 - 1.71 | Metlin 239 | Exp | Yes |
| Dihydroxyquinoline-β-glucuronide | [M+H]^+^ | C_15_H_15_NO_8_ | 2b | 338.0869 | 338.087 | 320.0765 | 302.0659 | 292.0816 | 162.055 | 320.0977 | 302.0807 | / | 162.0548 | 1.79 | HMDB0011658 | Theo | No |
| Phenylalanine | [M+H]^+^ | C_9_H_11_NO_2_ | 2a | 166.0849 | 166.0858 | 149.0591 | 131.0484 | 120.0806 | 103.0537 | 149.06 | 131.0492 | 120.0807 | 103.0559 | 2.1 | Metlin 28 | Exp | Yes |
| Porphobilinogen | [M+H]^+^ | C_10_H_14_N_2_O_4_ | 2b | 227.1025 | 227.11 | 210.0876 | 182.0926 | 165.0826 | 138.0645 | 210.0745 | 182.0876 | / | / | 2.28 | Metlin 76 | Exp | No |
| Dihydrocaffeic acid glucuronic acid | [M+H]^+^ | C_15_H_18_O_10_ | 2b | 359.0874 | 359.0973 | 341.0867 | 323.0761 | 183.0652 | 165.0546 | / | 323.0755 | 183.0666 | 165.055 | 2.38 | HMDB0041720 | Theo | No |
| 2-indole-carboxylic acid (glucuronide)* | [M+H]^+^ | C_9_H_7_NO_2_ | 2b | 162.0556 | 162.0555 | 144.0449 | 134.0242 | 118.0293 | 116.05 | 144.0438 | / | / | 116.0508 | 2.76 | HMDB0002285 | Theo | No |
| Xanthurenic acid (glucuronide)* | [M+H]^+^ | C_10_H_7_NO_4_ | 2b | 206.0441 | 206.0442 | 188.0336 | 178.0462 | 160.0382 | 132.0435 | 188.0238 | 178.0486 | 160.0389 | 132.0482 | 2.77 | Metlin 5481* | Theo | No |
| 4-(2-aminophenyl)2,4-dioxobutanoate | [M+H]^+^ | C_10_H_9_NO_4_ | 2b | 208.0613 | 208.061 | 190.0504 | 164.0712 | 162.0555 | 120.0449 | 190.0499 | 164.0658 | 162.0555 | 120.047 | 3.38 | HMDB0000978 | Theo | No |
| Hippuric acid | [M+H]^+^ | C_9_H_9_NO_3_ | 2a | 180.0667 | 180.0672 | 139.0574 | 121.1007 | 105.0338 | 77.0394 | / | 121.1048 | 105.0348 | 77.0402 | 3.58 | Metlin 1301 | Exp | Yes |
| Hydroxyprolyl-methionine | [M+H]^+^ | C_10_H_18_N_2_O_4_S | 2b | 263.1037 | 263.106 | 245.0954 | 227.0849 | 217.1005 | 199.09 | 245.0905 | 227.0887 | 217.0969 | 199.30889 | 3.62 | HMDB0028869 |  | No |
| Biotin | [M+H]^+^ | C_10_H_16_N_2_O_3_S | 2a | 245.0923 | 245.0956 | 227.0849 | 184.0819 | 167.0533 | 166.0685 | 227.0839 | / | / | / | 3.7 | Metlin 243 | Exp | Yes |
| Phenylacetylglycine | [M+H]^+^ | C_10_H_11_NO_3_ | 2a | 194.0813 | 194.0808 | 148.0745 | 120.0814 | 91.0546 | 76.0398 | 148.0755 | 120.081 | 91.0549 | 76.0404 | 4.04 | Metlin 4237 | Exp | Yes |
| 5-hydroxyindoleacetic acid | [M+H]^+^ | C_10_H_9_NO_3_ | 2b | 192.0663 | 192.0637 | 146.0599 | 110.0086 | 68.9833 | 66.0208 | 146.0607 | 110.0106 | / | / | 4.13 | Metlin 2975 | Exp | No |
| Hexanoylglycine | [M+H]^+^ | C_8_H_15_NO_3_ | 2b | 174.1123 | 174.1125 | 156.1019 | 128.107 | 99.0804 | 76.0393 | 156.1024 | 128.1125 | 99.082 | 76.0385 | 4.68 | HMDB0000701 | Exp | No |
| Cinnamoylglycine | [M+H]^+^ | C_11_H_11_NO_3_ | 2b | 206.0479 | 206.0464 | 188.0373 | 160.0378 | 131.0488 | 103.0547 | 188.0381 | / | 131.0507 | 103.0556 | 5.34 | Metlin 34534 | Exp | No |
| Tetranor-5-NO2-CLA | [M+Na]^+^ | C_14_H_23_NO_4_ | 2b | 292.1532 | 292.1519 | 270.17 | 252.1594 | / | / | 270.1711 | 252.1606 | 206.1535 | 177.1279 | 5.7 | LMFA01120010 | Theo | No |
| Octenoylglycine | [M+H]^+^ | C_10_H_17_NO_3_ | 2b | 200.13 | 200.1281 | 183.1016 | 182.1176 | 165.091 | 154.1226 | / | 182.1196 | 165.0939 | 154.1247 | 6.2 - 6.31 | HMDB0094801 | Theo | No |

**A neutral loss of 176 Da was detected compared to the [M+H]+ adduct but the annotation herein is reported according the aglycone.*

**Table S3. Metabolite annotations putatively performed on the features found to be significantly affected by the inhibition of the bacterial β-glucuronidase in urine samples in UPLC-MS ESI- mode.** All the m/z reported correspond to [M+H]^+^ adducts and the level of annotations are reported according the levels of confidence described in the experimental section.

| **putative ID** | **Adduct** | **formula** | **ID level** | ***m/z*** | **Calculated mass** | **frag 1 theo** | **frag 2 theo** | **frag 3 theo** | **frag 4 theo** | **frag 1 obs** | **frag 2 obs** | **frag 3 obs** | **frag 4 obs** | **RT (mins)** | **DB** | **In silico / exp** | **NPC DB** |
| --- | --- | --- | --- | --- | --- | --- | --- | --- | --- | --- | --- | --- | --- | --- | --- | --- | --- |
|  |  |  |  |  |  |  |  |  |  |  |  |  |  |  |  |  |  |
| 5-hydroxylysine | [M-H]^-^ | C_6_H_14_N_2_O_3_ | 2b | / | 161.0926 | 144.0661 | 143.0821 | 130.0504 | 117.1028 | 144.0651 | 143.084 | / | / | 0.55 | HMDB0000450 | Theo | No |
| Taurine | [M-H]^-^ | C_2_H_7_NO_3_S | 2a | 124.0069 | 124.0062 | 106.9814 | 94.9808 | 80.9651 | 79.957 | 106.9842 | 94.9783 | 80.9629 | 79.9568 | 0.56 | Metlin 31 | Exp | Yes |
| Glycerol-phosphate | [M+Br]^-^ | C_3_H_9_O_6_P | 2a | 251.0491 | 251.047 | 171.0139 | 114.943 | 86.9067 | 71.045 | 171.0058 | / | / | / | 0.58 | HMDB0000126 | Exp | Yes |
| Glutamate | [M-H]^-^ | C_5_H_9_NO_4_ | 2a | 146.0463 | 146.0452 | 128.035 | 102.056 | 85.0293 | / | 128.0353 | / | 85.0279 | / | 0.58 | Metlin 19 | Exp | Yes |
| *N6,N6,N6-*trimethyl-L-lysine | [M-H]^-^ | C_9_H_20_N_2_O_2_ | 2a | / | 187.1452 | 130.0874 | 128.0717 | 114.0561 | 74.0248 | / | 128.0698 | 114.0509 | 74.0256 | 0.61 | HMDB0001325 | Theo | Yes |
| Allantoin | [M-H]^-^ | C_4_H_6_N_4_O_3_ | 2a | 157.0355 | 157.0344 | 140.0056 | 114.0292 | 97.0034 | 71.0251 | 140.0068 | 114.0296 | 97.0027 | / | 0.63 | Metlin 89 | Exp | Yes |
| *N-*acetylneuraminic acid | [M-H]^-^ | C_11_H_19_NO_9_ | 2b | 308.0989 | 308.0983 | 290.0875 | 170.0453 | 98.0607 | 87.0091 | 290.0854 | 170.0472 | 98.0619 | 87.0092 | 0.64 | Metlin 5739 | Exp | No |
| Glycyl-aspartate | [M-H]^-^ | C_6_H_10_N_2_O_5_ | 2b | 189.0518 | 189.0517 | 171.0411 | 145.0619 | 132.0302 | 127.0513 | 171.0398 | / | 132.0383 | / | 0.68 | HMDB0028837 | Theo | No |
| Arabinose | [M-H]^-^ | C_5_H_10_O_5_ | 2a | 149.0452 | 149.0425 | 131.0599 | 113.0226 | 101.0233 | 89.0237 | 131.0348 | 113.0242 | 101.0239 | 89.0246 | 0.68 | Metlin 65497 | Exp | Yes |
| Sucrose | [M-H]^-^ | C_12_H_22_0_12_ | 2a | 341.1071 | 341.01085 | 179.0553 | 161.0444 | 143.0342 | 119.0341 | 179.055 | 161.0385 | 143.033 | 119.033 | 0.7 | Metlin 137 | Exp | Yes |
| *N-*acetylhistidine | [M-H]^-^ | C_8_H_11_N_3_O_3_ | 2b | 196.0721 | 196.0722 | 178.0617 | 154.0617 | 152.0824 | 134.0718 | / | 154.0614 | 152.0831 | 134.068 | 0.71 | HMDB0032055 | Theo | No |
| 4-guanidino butanoic acid | [M-H]^-^ | C_5_H_11_N_3_O_2_ | 2b | 144.0767 | 144.0782 | 127.051 | 102.0555 | 84.0448 | 83.0612 | 127.0554 | 102.0576 | / | 83.063 | 0.94 | Metlin 4155 | Exp | No |
| *N-*acetylglutamic acid | [M-H]^-^ | C_7_H_11_NO_5_ | 2b | 188.0561 | 188.0553 | 170.0446 | 144.0654 | 128.0345 | 102.0555 | 170.0468 | 144.0658 | 128.0352 | 102.0566 | 1.38 | Metlin 3325 | Exp | No |
| Succinic acid | [M-H]^-^ | C_4_H_6_O_4_ | 2a | 117.0189 | 117.019 | 99.0086 | 73.03 | 55.019 | / | 99.008 | 73.0279 | / | / | 1.41 | Metlin 114 | Exp | Yes |
| Tyrosine | [M-H]^-^ | C_9_H_11_NO_3_ | 2b | 180.0679 | 180.0654 | 163.0392 | 119.0502 | 93.0346 | 72.0089 | 163.041 | 119.053 | 93.0433 | / | 1.43 | Metlin 34 | Exp | No |
| Deoxyuridine | [M-H]^-^ | C_9_H_12_N_2_O_5_ | 2a | 227.0672 | 227.066 | 184.0593 | 136.0372 | 111.0195 | 94.03 | 184.0613 | 136.042 | 111.009 | 94.0301 | 1.62 | Metlin 91 | Exp | Yes |
| 4-pyridoxate | [M-H]^-^ | C_8_H_9_NO_4_ | 2b | 182.0457 | 182.0448 | 138.0552 | 123.0315 | 120.0442 | 108.0446 | 138.0557 | 123.0329 | 120.0451 | 108.046 | 1.75 | Metlin 239 | Exp | No |
| Dihydroxyquinoline-β-glucuronide | [M-H]^-^ | C_15_H_15_NO_8_ | 2b | 336.0726 | 336.0725 | 318.0619 | 292.087 | 290.067 | 160.0404 | 318.0638 | / | / | 160.0398 | 1.99 | HMDB0011658 | Theo | No |
| Porphobilinogen | [M-H]^-^ | C_10_H_14_N_2_O_4_ | 2b | 225.08872 | 225.0872 | 181.097 | 163.0864 | 152.071 | 120.0816 | 181.0954 | 163.0875 | / | / | 2.32 | Metlin 76 | Exp | No |
| Dihydrocaffeic acid glucuronic acid | [M-H]^-^ | C_15_H_18_O_10_ | 2b | 357.0825 | 357.0827 | 339.0722 | 181.0506 | 163.0401 | 117.0193 | 339.0734 | 181.049 | 163.0456 | 117.0192 | 2.42 | HMDB0041720 | Theo | No |
| *P-*hydroxyphenyllactic acid | [M-H]^-^ | C_9_H_10_O_4_ | 2a | 181.0495 | 181.0496 | 163.0385 | 135.044 | 119.0505 | 72.9928 | 163.0412 | 135.0443 | 119.05 | / | 2.72 | Metlin 34515* | Exp | Yes |
| 2-indole-carboxylic acid (glucuronide)* | [M-H]^-^ | C_9_H_7_NO_2_ | 2b | 160.0403 | 160.0399 | 142.0293 | 116.05 | 114.0344 | / | / | 116.053 | / | / | 2.76 | HMDB0002285 | Theo | No |
| Xanthurenic acid (glucuronide)* | [M-H]^-^ | C_10_H_7_NO_4_ | 2b | 204.0296 | 204.029 | 160.0391 | 132.0459 | 116.049 | / | 160.0397 | 132.0432 | 116.0495 | / | 2.85 | Metlin 5481* | Theo | No |
| 5-hydroxyindoleacetic acid | [M-H]^-^ | C_10_H_9_NO_3_ | 2a | 190.0504 | 190.0407 | 146.0602 | 144.044 | 131.0382 | / | 146.0606 | 144.0442 | 131.0368 | / | 3.18 - 3.38 | Metlin 2975 | Exp | Yes |
| Phenyl glucuronide | [M-H]^-^ | C_12_H_14_O_7_ | 2b | 269.0659 | 269.0667 | 251.0555 | 175.0243 | 129.0189 | 113.0241 | 251.0556 | 175.024 | 129.0551 | 113.0245 | 3.28 | Metlin 1825 | Exp | No |
| 4-(2-aminophenyl)2,4-dioxobutanoate | [M-H]^-^ | C_10_H_9_NO_4_ | 2b | 206.0452 | 206.0453 | 188.0348 | 162.0555 | 160.0399 | 145.029 | 188.0351 | 162.0555 | 160.0398 | 145.0452 | 3.38 | HMDB0000978 | Theo | No |
| *P-*coumaric acid sulfate | [M-H]^-^ | C_9_H_8_O_6_S | 2b | 242.9965 | 242.9969 | 224.9863 | 199.0071 | 163.0401 | 145.0295 | / | 199.0062 | 163.0396 | / | 3.49 | HMDB0125166 | Theo | No |
| Pimelic acid | [M-H]^-^ | C_7_H_12_O_4_ | 2a | 159.0663 | 159.0647 | 141.0545 | 115.0755 | 97.0656 | 95.0502 | / | 115.0766 | 97.0648 | 95.0466 | 3.51 | Metlin 3280 | Exp | Yes |
| Indoxylsulfuric acid | [M-H]^-^ | C_8_H_7_NO_4_S | 2a | 212.0011 | 212.0019 | 132.0448 | 80.9653 | 79.9576 | / | 132.045 | 80.963 | 79.954 | / | 3.53 | Metlin 252 | Exp | Yes |
| Hippuric acid | [M-H]^-^ | C_9_H_9_NO_3_ | 2a | 178.053 | 178.0503 | 134.0607 | 132.0449 | 77.0399 | 56.0139 | 134.053 | 132.0449 | 77.0387 | / | 3.61 | Metlin 1301 | Exp | Yes |
| Ferulic acid 4-O-slufate | [M-H]^-^ | C_10_H_10_O_7_S | 2b | 273.0073 | 273.0074 | 254.9969 | 229.0176 | 193.0506 | 175.0401 | 254.9608 | 229.0183 | 193.0503 | 175.041 | 3.67 | HMDB0029200 | Theo | No |
| Phenylacetylglycine | [M-H]^-^ | C_10_H_11_NO_3_ | 2a | 192.0661 | 192.0631 | 91.054 | 74.0244 | / | / | 91.0561 | 74.0246 | / | / | 4.04 | Metlin 4237 | Exp | Yes |
| *P-*cresol-glucuronide | [M-H]^-^ | C_13_H_16_O_7_ | 2b | 283.082 | 283.0818 | 265.0712 | 239.0919 | 175.0243 | 107.0497 | 265.0717 | 239.0977 | 175.0226 | 107.05 | 4.35 | HMDB0011686 | Theo | No |
| *P-*cresol-sulfate | [M-H]^-^ | C_7_H_8_O_4_S | 2b | 187.0068 | 187.0065 | 160.9909 | 107.0497 | 105.043 | 80.9646 | / | 107.0499 | 105.0324 | 80.9641 | 4.46 | HMDB0011635 | Theo | No |
| Hexanoylglycine | [M-H]^-^ | C_8_H_15_NO_3_ | 2b | 172.0968 | 172.0979 | 154.0874 | 128.1081 | 126.0924 | 97.0659 | / | / | / | / | 4.68 | HMDB0000701 | Theo | No |
| Cinnamoylglycine | [M-H]^-^ | C_11_H_11_NO_3_ | 2b | 204.0662 | 204.0656 | 160.0758 | 130.0654 | 117.0693 | 103.0545 | 160.0773 | 130.0654 | 117.0722 | 103.0559 | 5.34 | Metlin 34534 | Exp | No |
| Tetranor-5-NO2-CLA | [M-H]^-^ | C_14_H_23_NO_4_ | 2b | 268.1545 | 268.1554 | 206.1548 | 168.0663 | 154.0507 | 112.0403 | 206.1525 | / | / | / | 5.73 | LMFA01120010 | Exp | No |
| Octenoylglycine | [M-H]^-^ | C_10_H_17_NO_3_ | 2b | 198.1126 | 198.1136 | 181.087 | 180.103 | 154.1237 | 152.108 | 181.0918 | / | 154.1228 | / | 6.2 - 6.31 | HMDB0094801 | Theo | No |

**A neutral loss of 176 Da was detected compared to the [M-H]- adduct but the annotation herein is reported according the aglycone.*

**Table S4. Metabolite annotations putatively performed on the features found to be significantly affected by the inhibition of the bacterial β-glucuronidase in colon contents in UPLC-MS ESI+ mode.** All the m/z reported correspond to [M+H]^+^ adducts and the level of annotations are reported according the levels of confidence described in the experimental section.

| **putative ID** | **formula** | **ID level** | ***m/z*** | **Calculated mass** | **frag 1 theo** | **frag 2 theo** | **frag 3 theo** | **frag 4 theo** | **frag 1 obs** | **frag 2 obs** | **frag 3 obs** | **frag 4 obs** | **RT (mins)** | **DB** | **In silico / exp** | **NPC DB** |
| --- | --- | --- | --- | --- | --- | --- | --- | --- | --- | --- | --- | --- | --- | --- | --- | --- |
|  |  |  |  |  |  |  |  |  |  |  |  |  |  |  |  |  |
| Glutamine | C_5_H_10_N_2_O_3_ | 2a | 147.0765 | 147.0762 | 130.0495 | 102.0547 | 101.071 | 84.0448 | 130.0504 | 102.0561 | 101.0719 | 84.0451 | 0.57 | Metlin 18 | Exp | Yes |
| Glutamate | C_5_H_9_NO_4_ | 2a | 148.0609 | 148.0607 | 130.0501 | 102.0548 | 84.0446 | / | 130.0503 | 102.0557 | 84.0449 | / | 0.59 | Metlin 19 | Exp | Yes |
| Citrulline | C_6_H_13_N_3_O_3_ | 2a | 176.103 | 176.1019 | 159.0757 | 116.0715 | 115.087 | 113.0706 | 159.0766 | 116.0715 | 115.0869 | 113.0706 | 0.61 | Metlin 16 | Exp | Yes |
| Glucosamine phosphate | C_7_H_13_N_3_O_4_ | 2b | 260.0458 | 260.053 | 162.0761 | 146.0812 | 144.0655 | 98.9842 | 162.0765 | 146.0819 | 144.0652 | 98.9751 | 0.63 | Metlin 3390 | Theo | No |
| Formino-glutamic acid | C_6_H_10_N_2_O_4_ | 2a | 175.0715 | 175.0719 | 157.0613 | 148.061 | 140.0348 | 129.0188 | 157.0604 | 148.0609 | / | / | 0.64 | HMDB0000854 | Theo | Yes |
| 5-aminopentanoic acid | C_5_H_11_NO_2_ | 2a | 118.0867 | 118.0864 | 101.0602 | 100.076 | 82.0657 | 56.0504 | 101.0608 | 100.0764 | 82.0665 | 56.0505 | 0.67 | Metlin 6902 | Exp | Yes |
| Glycyl-proline | C_7_H_12_N_2_O_3_ | 2a | 173.0921 | 173.0915 | 155.081 | 127.0863 | 116.0706 | 70.0655 | 155.0819 | 127.0868 | 116.071 | 70.0662 | 0.68 | Metlin 4185 | Exp | Yes |
| Leucine | C_6_H_13_NO_2_ | 2b | 132.1022 | 132.1016 | 86.0969 | 73.067 | 69.07 | 55.0538 | 86.0973 | / | / | / | 0.96 | Metlin 24 | Exp | No |
| Uridine | C_9_H_12_N_2_O_6_ | 2a | 133.0491 | 133.0489 | 115.0372 | 113.0349 | 97.0298 | 85.0289 | 115.0352 | 113.0354 | 97.0285 | / | 1.41 | Metlin 90 | Exp | No |
| Tyrosine | C_9_H_11_NO_3_ | 2b | 182.0821 | 182.0864 | 165.0548 | 147.0451 | 136.0753 | 123.0424 | 165.0553 | 147.0438 | 136.0764 | 123.0444 | 1.43 | Metlin 34 | Exp | No |
| Guanine | C_5_H_5_N_5_O | 2b | 152.0559 | 152.0566 | 135.0299 | 110.0355 | 107.0357 | 82.0409 | 135.0297 | 110.0348 | 107.0487 | 82.043 | 1.63 | Metlin 315 | Exp | No |
| Hypoxanthine | C_5_H_4_N_4_O | 2b | 137.0458 | 137.0455 | 119.0351 | 110.0348 | 94.0403 | 82.0406 | 119.0389 | 110.0356 | 94.0412 | 82.0404 | 1.64 | Metlin 83 | Exp | No |
| Deoxyguanosine | C_10_H_13_N_5_O_4_ | 2a | 268.1074 | 268.1031 | 152.0558 | 135.0291 | 117.0535 | 99.0437 | 152.0572 | 135.0323 | 117.0522 | 99.0424 | 1.72 | Metlin 3395 | Exp | Yes |
| Proline | C_5_H_9_NO_2_ | 2b | 116.0702 | 116.0708 | 70.0659 | 68.0506 | / | / | 70.0662 | 68.0513 | / | / | 1.77 | Metlin 29 | Exp | No |
| Pro-Tyr | C_14_H_18_N_2_O_4_ | 2b | 279.1338 | 279.1329 | 233.1282 | 182.0807 | 70.0651 | / | 233.1484 | 182.0793 | 70.0667 | / | 2.17 - 2.43 | Metlin 23717 | Exp | No |
| Leu-Val | C_11_H_22_N_2_O_3_ | 2b | 231.1703 | 231.1671 | 185.1613 | 118.0843 | 86.0955 | 72.0803 | 185.1661 | 118.0859 | 86.0965 | 72.0817 | 2.45 | Metlin 23890 | Exp | No |
| Val-Leu | C_11_H_22_N_2_O_3_ | 2b | 213.158 | 213.1666 | 185.1618 | 132.1 | 86.0944 | 72.0796 | 185.165 | 132.1016 | 86.097 | 72.0816 | 2.59 | Metlin 23950 | Exp | No |
| Pro-Ile | C_11_H_20_N_2_O_3_ | 2a | 229.1547 | 229.1523 | 211.1388 | 183.1473 | 86.093 | 70.0645 | / | / | 86.0968 | 70.0667 | 2.67 | Metlin 23879 | Exp | Yes |
| Indole-3-carbaldehyde | C_9_H_7_NO | 2b | 146.0612 | 146.0589 | 118.0642 | 91.0528 | 65.0379 | / | 118.0656 | 91.0542 | / | / | 2.69 | HMDB0029737 | Exp | No |
| Serotonin | C_10_H_12_N_2_O | 2b | 177.1031 | 177.102 | 160.0752 | 142.0648 | 132.0806 | 115.0541 | / | 142.0652 | 132.0799 | 155.0546 | 2.78 | Metlin 23 | Exp | No |
| Indole acrylic acid | C_11_H_9_NO_2_ | 2b | 188.0712 | 188.0698 | 170.06 | 146.0602 | 142.0645 | 115.0534 | 170.0628 | 146.06 | 142.0655 | 115.0547 | 2.8 | Metlin 5702 | Exp | No |
| 4-(2-aminophenyl)2,4-dioxobutanoate | C_10_H_9_NO_4_ | 2b | 208.0592 | 208.061 | 190.0504 | 164.0712 | 162.0555 | 120.0449 | 190.0486 | / | 162.0555 | 120.0443 | 3.38 | HMDB0000978 | Theo | No |
| Gly-Lys-Tyr | C_17_H_26_N_4_O_5_ | 2b | 364.2003 | 367.1946 | 332.1599 | 310.1715 | 186.1209 | 129.1006 | 332.1543 | / | 186.1243 | 129.1013 | 3.53 | Metlin 20072 | Exp | No |
| Ser-Ile-Leu | C_15_H_29_N_3_O_5_ | 2b | 332.2135 | 332.225 | 201.1272 | 173.1321 | 132.1058 | 86.0985 | 201.1245 | 173.1293 | 132.1023 | 86.0974 | 3.57 | Metlin 22825 | Exp | No |
| Phe-Leu | C_15_H_22_N_2_O_3_ | 2b | / | 279.1687 | 233.1623 | 132.0994 | 120.0792 | 86.0941 | 233.1669 | 132.0986 | 120.081 | 86.0981 | 3.72 | Metlin 23831 | Exp | No |
| Pro-Trp | C_16_H_19_N_3_O_3_ | 2b | 302.1563 | 302.1487 | 205.095 | 188.0686 | 181.1006 | 70.0653 | 205.0879 | 188.0699 | / | 70.0654 | 3.94 | Metlin 24006 | Exp | No |
| Arg-Gln-Arg | C_17_H_34_N_10_O_5_ | 2b | 459.2759 | 459.2802 | 442.2558 | 303.1753 | 286.1471 | 215.3198 | 442.2702 | / | 286.144 | / | 4.3 | Metlin 19907 | Exp | No |
| Trp-Ole or Trp-Leu | C_17_H_23_N_3_O_3_ | 2b | 318.1697 | 318.1799 | 301.153 | 273.1581 | 159.0906 | 144.0805 | 301.1552 | 273.1696 | / | 144.0855 | 5.26 | Metlin 23956 | Exp | No |
| Tetranor-23-oxo-hydroxyvitamin D3 | C_23_H_34_O_3_ | 2b | 359.2561 | 359.2581 | 341.2475 | 323.2369 | 219.1743 | 139.0754 | 341.248 | 323.1792 | 219.1415 | / | 8.73 | HMDB0060114 | Theo | No |
| Nutriacholic acid | C_24_H_38_O_4_ | 2b | / | 391.2843 | 373.2737 | 355.2632 | 345.2788 | 327.2682 | 373.2695 | 355.262 | 345.2475 | / | 8.93 | HMDB0000467 | Theo | No |
| Cervonoyl ethanolamide | C_24_H_36_O_3_ | 2b | 373.2722 | 373.2737 | 355.2632 | 329.2475 | 311.2369 | 103.039 | 355.2637 | 329.2156 | / | / | 9.11 | HMDB0013627 | Theo | No |
| Cervonic acid ethyl ester | C_24_H_36_O_2_ | 2b | 357.279 | 357.2777 | 311.2355 | 215.1773 | 119.0845 | 175.146 | 311.1736 | 215.1841 | 119.0849 | 175.1455 | 9.58 | Metlin 62947 | Exp | No |
| Pa(10:0/8:0) | C_21_H_41_O_8_P | 2b | 453.2591 | 453.2612 | 435.2506 | 373.2949 | 355.2843 | 213.1632 | 435.2499 | 373.2746 | 355.2639 | 213.1849 | 9.71 | HMDB0114778 | Theo | No |
| Tetracosahexanoic acid | C_24_H_36_O_2_ | 2b | 357.2791 | 357.2788 | 339.2682 | 321.2577 | 311.2733 | 297.2577 | 339.265 | 321.2613 | / | 293.2396 | 9.95 | HMDB0002007 | Theo | No |

**Table S5. Metabolite annotations putatively performed on the features found to be significantly affected by the inhibition of the bacterial β-glucuronidase in colon tissues in UPLC-MS ESI+ mode.** All the m/z reported correspond to [M+H]^+^ adducts and the level of annotations are reported according the levels of confidence described in the experimental section.

| **putative ID** | **formula** | **ID level** | ***m/z*** | **Calculated mass** | **frag 1 theo** | **frag 2 theo** | **frag 3 theo** | **frag 4 theo** | **frag 1 obs** | **frag 2 obs** | **frag 3 obs** | **frag 4 obs** | **RT (mins)** | **DB** | **In silico / exp** | **NPC DB** |
| --- | --- | --- | --- | --- | --- | --- | --- | --- | --- | --- | --- | --- | --- | --- | --- | --- |
|  |  |  |  |  |  |  |  |  |  |  |  |  |  |  |  |  |
| *NG,NG-*dimethyl-L-arginine | C_8_H_18_N_4_O_2_ | 2a | 203.1603 | 203.1497 | 158.1285 | 116.0708 | 88.0875 | 70.0659 | / | 116.0828 | / | 70.0741 | 0.78 | Metlin 6891 | Exp | Yes |
| Tyrosine | C_9_H_11_NO_3_ | 2b | 182.2005 | 182.0864 | 165.0548 | 147.0451 | 136.0753 | 123.0424 | 165.0721 | 147.0603 | 136.0902 | 123.0567 | 1.43 | Metlin 34 | Exp | No |
| 4-(2-aminophenyl)2,4-dioxobutanoate | C_10_H_9_NO_4_ | 2b | 208.0849 | 208.061 | 190.0504 | 164.0712 | 162.0555 | 120.0449 | 190.0728 | / | 162.0619 | 120.0952 | 3.38 | HMDB0000978 | Theo | No |
| *N-*acetylleucine | C_8_H_15_NO_3_ | 2a | 174.0926 | 174.1147 | 156.1035 | 132.1034 | 128.1088 | 86.0987 | 156.098 | 132.1169 | 128.0802 | 86.1016 | 4.23 | Metlin 43866 | Exp | Yes |
| Glycocholate | C_26_H_43_NO_6_ | 2a | 466.3685 | 466.3151 | 448.3056 | 430.2936 | 412.2836 | 337.2524 | 448.2846 | 430.2847 | 412.267 | 337.2895 | 8.46 | Metlin 202 | Exp | Yes |
| Nutriacholic acid | C_24_H_38_O_4_ | 2b | 391.2386 | 391.2843 | 373.2737 | 355.2632 | 345.2788 | 327.2682 | 373.3144 | 355.2968 | / | 327.258 | 8.93 | HMDB0000467 | Theo | No |
| Tetracosahexanoic acid | C_24_H_36_O_2_ | 2b | 357.3146 | 357.2788 | 339.2682 | 321.2577 | 311.2733 | 297.2577 | 339.3076 | 321.2757 | 311.2566 | 297.271 | 9.95 | HMDB0002007 | Theo | No |

**Table S6. Biochemical origins of the metabolites observed to differ in the urine, colon contents and colon tissues between control and UNC10201652 treated animals.**

| **Metabolite** | **Tissue** | **Change with treatment** | **Origin** |
| --- | --- | --- | --- |
| Leucine | Urine | Decrease | Exogenous |
| Sucrose | Urine | Decrease | Exogenous |
| Arabinose | Urine | Increase | Exogenous |
| Ferulic acid 4-0-sulfate | Urine | Decrease | Host |
| 2-indole-carboxylic acid (glucuronide) | Urine | Increase | Host |
| 3-hydroxyanthranilic acid | Urine | Decrease | Host |
| Glycyl-aspartate | Urine | Decrease | Host |
| Glycyl-proline | Urine | Increase | Host |
| Hexanoylglycine | Urine | Decrease | Host |
| Hydroxyprolyl-methionine | Urine | Increase | Host |
| *N-*acetylglutamine | Urine | Increase | Host |
| *N-*acetylspermidine | Urine | Decrease | Host |
| *N6,N6,N6*-trimethyl-L-lysine | Urine | Decrease | Host |
| Octenoylglycine | Urine | Decrease | Host |
| Porphobilinogen | Urine | Decrease | Host |
| *S-*adenosylmethionine | Urine | Increase | Host |
| Xanthurenic acid (glucuronide) | Urine | Decrease | Host |
| Deoxyuridine | Urine | Decrease | Host |
| Glycerol-phosphate | Urine | Increase | Host |
| *N-*acetylneuraminic acid | Urine | Decrease | Host |
| *p-*Coumaric acid sulfate | Urine | Decrease | Host |
| Phenyl glucuronide | Urine | Increase | Host |
| Carnitine | Urine | Increase | Host and Exogenous |
| Creatine | Urine | Decrease | Host and Exogenous |
| Taurine | Urine | Decrease | Host and Exogenous |
| Trigonelline | Urine | Decrease | Host and exogenous |
| 4-guanidinobutanoic acid | Urine | Decrease | Host and Exogenous |
| Trans-Aconitate | Urine | Decrease | Host and Exogenous |
| 4-(2-aminophenyl)2,4-dioxobutanoate | Urine | Increase | Host and Microbial |
| 4-pyridoxate | Urine | Increase | Host and Microbial |
| 5-hydroxyindoleacetic acid | Urine | Increase | Host and Microbial |
| Citrulline | Urine | Decrease | Host and Microbial |
| *N-*acetyllysine | Urine | Increase | Host and Microbial |
| *N-*acetylneuraminic acid | Urine | Decrease | Host and Microbial |
| *N-*acetylputrescine | Urine | Increase | Host and Microbial |
| *P-*hydroxyphenyllactic acid | Urine | Decrease | Host and Microbial |
| Pimelic acid | Urine | Decrease | Host and Microbial |
| Succinic acid | Urine | Increase | Host and Microbial |
| Creatinine | Urine | Decrease | Host, Exogenous and Microbial |
| Glutamate | Urine | Decrease | Host, Exogenous and Microbial |
| Histidine | Urine | Decrease | Host, Exogenous and Microbial |
| *N-*acetylglutamic acid | Urine | Increase | Host, Exogenous and Microbial |
| Tyrosine | Urine | Decrease | Host, Exogenous and Microbial |
| Uridine | Urine | Decrease | Host, Exogenous and Microbial |
| Allantoin | Urine | Increase | Host, Exogenous and Microbial |
| Orotate | Urine | Increase | Host, Exogenous and Microbial |
| 5-aminopentanoic acid | Urine | Decrease | Microbial |
| Biotin | Urine | Decrease | Microbial |
| *NG,NG-*dimethyl-arginine | Urine | Decrease | Microbial |
| Trimethylamine-*N-*oxide (TMA) | Urine | Decrease | Microbial |
| Lysine | Urine | Increase | Microbial and Exogenous |
| Thiamine | Urine | Decrease | Microbial and Exogenous |
| Cinnamoylglycine | Urine | Increase | Microbial-Host co-metabolite |
| Dihydrocaffeic acid glucuronic acid | Urine | Decrease | Microbial-Host co-metabolite |
| Hippuric acid | Urine | Increase | Microbial-Host co-metabolite |
| Phenylacetylglycine | Urine | Increase | Microbial-Host co-metabolite |
| 3-indoxyl sulfate | Urine | Increase | Microbial-Host co-metabolite |
| Dihydroxyquinoline-β-glucuronide | Urine | Decrease | Microbial-Host co-metabolite |
| *p-C*resyl-glucuronide | Urine | Increase | Microbial-Host co-metabolite |
| *p-C*resyl-sulfate | Urine | Increase | Microbial-Host co-metabolite |
| Trimethylamine-*N-*oxide (TMAO) | Urine | Increase | Microbial-Host co-metabolite |
| Leucine | Colon contents | Decrease | Exogenous |
| Cervonic acid ethyl ester | Colon contents | Decrease | Exogenous |
| Cervonoyl Ethanolamide | Colon contents | Decrease | Host |
| Pro-Trp | Colon contents | Decrease | Host |
| Pro-Ile | Colon contents | Decrease | Host |
| Trp-Ile or Trp-Leu | Colon contents | Decrease | Host |
| Arg-Gln-Arg | Colon contents | Decrease | Host |
| Pro-Tyr | Colon contents | Decrease | Host |
| Phe-Leu | Colon contents | Decrease | Host |
| PA(10:0/8:0) | Colon contents | Decrease | Host |
| Gly-Lys-Tyr | Colon contents | Decrease | Host |
| Ser-Ile-Leu | Colon contents | Decrease | Host |
| Val-Leu | Colon contents | Decrease | Host |
| Glycyl-proline | Colon contents | Decrease | Host |
| Leu-Val | Colon contents | Decrease | Host |
| Tetracosahexanoic acid | Colon contents | Decrease | Host and Exogenous |
| Serotonin | Colon contents | Decrease | Host and Microbial |
| Hypoxanthine | Colon contents | Decrease | Host and Microbial |
| Guanine | Colon contents | Decrease | Host and Microbial |
| Citrulline | Colon contents | Decrease | Host and Microbial |
| Glucosamine phosphate | Colon contents | Increase | Host and Microbial |
| 4-(2-aminophenyl)2,4-dioxobutanoate | Colon contents | Increase | Host and Microbial |
| Glutamate | Colon contents | Decrease | Host, Microbial and Exogenous |
| Uridine | Colon contents | Decrease | Host, Microbial and Exogenous |
| Tyrosine | Colon contents | Decrease | Host, Microbial and Exogenous |
| Proline | Colon contents | Decrease | Host, Microbial and Exogenous |
| Indole-3-carbaldehyde | Colon contents | Decrease | Microbial and Exogenous |
| 5-aminopentanoic acid | Colon contents | Decrease | Microbial |
| 3-indoxyl sulfate | Colon contents | Decrease | Microbial-Host co-metabolite |
| Nutriacholic acid | Colon contents | Increase | Microbial-Host co-metabolite |
| 4-(2-aminophenyl)2,4-dioxobutanoate | Colon tissue | Increase | Host and Microbial |
| Glycocholate | Colon tissues | Increase | Host |
| *N-*acetylleucine | Colon tissues | Increase | Host |
| *NG,NG-*dimethyl-L-arginine | Colon tissues | Increase | Microbial |
| Nutriacholic acid | Colon tissues | Increase | Microbial-Host co-metabolite |
| Tyrosine | Colon tissues | Increase | Host, Microbial and Exogenous |
